# Single-cell and spatial transcriptome assays reveal heterogeneity in gliomas through stress responses and pathway alterations

**DOI:** 10.3389/fimmu.2024.1452172

**Published:** 2024-08-27

**Authors:** Zongze He, Zheng Liu, Qi Wang, Xingjian Sima, Wei Zhao, Chunmei He, Wenjie Yang, Han Chen, Bo Gong, Siyuan Song, Yi Wang

**Affiliations:** ^1^ Department of Neurosurgery, Sichuan Provincial People’s Hospital, University of Electronic Science and Technology of China, Chengdu, China; ^2^ Department of Medicine, Baylor College of Medicine, Houston, TX, United States; ^3^ Medical School, Tongji Medical College of Huazhong University of Science and Technology, Wuhan, China; ^4^ Center of Critical Care Medicine, Sichuan Provincial People’s Hospital, School of Medicine, University of Electronic Science and Technology of China, Chengdu, China; ^5^ Department of Otolaryngology, Chongqing General Hospital of the Chinese People’s Armed Police Force, Chongqing, China; ^6^ Department of Health Management, Sichuan Provincial People’s Hospital, University of Electronic Science and Technology of China, Chengdu, Sichuan, China; ^7^ The Key Laboratory for Human Disease Gene Study of Sichuan Province and Institute of Laboratory Medicine, Sichuan Provincial People’s Hospital, University of Electronic Science and Technology of China, Chengdu, Sichuan, China; ^8^ Department of Neuroscience, Baylor College of Medicine, Houston, TX, United States; ^9^ Clinical Immunology Translational Medicine Key Laboratory of Sichuan Province, Sichuan Academy of Medical Science and Sichuan Provincial People’s Hospital, Chengdu, China

**Keywords:** glioma, oligodendrocyte precursor cells (OPCs), oxidative stress response, single-cell RNA sequencing, spatial transcriptomics, heterogeneity

## Abstract

**Background:**

Glioma is a highly heterogeneous malignancy of the central nervous system. This heterogeneity is driven by various molecular processes, including neoplastic transformation, cell cycle dysregulation, and angiogenesis. Among these biomolecular events, inflammation and stress pathways in the development and driving factors of glioma heterogeneity have been reported. However, the mechanisms of glioma heterogeneity under stress response remain unclear, especially from a spatial aspect.

**Methods:**

This study employed single-cell RNA sequencing (scRNA-seq) and spatial transcriptomics (ST) to explore the impact of oxidative stress response genes in oligodendrocyte precursor cells (OPCs). Our analysis identified distinct pathways activated by oxidative stress in two different types of gliomas: high- and low- grade (HG and LG) gliomas.

**Results:**

In HG gliomas, oxidative stress induced a metabolic shift from oxidative phosphorylation to glycolysis, promoting cell survival by preventing apoptosis. This metabolic reprogramming was accompanied by epithelial-to-mesenchymal transition (EMT) and an upregulation of stress response genes. Furthermore, SCENIC (Single-Cell rEgulatory Network Inference and Clustering) analysis revealed that oxidative stress activated the AP1 transcription factor in HG gliomas, thereby enhancing tumor cell survival and proliferation.

**Conclusion:**

Our findings provide a novel perspective on the mechanisms of oxidative stress responses across various grades of gliomas. This insight enhances our comprehension of the evolutionary processes and heterogeneity within gliomas, potentially guiding future research and therapeutic strategies.

## Introduction

Gliomas are the most frequent form of central nervous system (CNS) tumor (1). China is one of the top three countries in terms of CNS cancer cases (including glioma) and fatalities (2, 3). In the United States, the average incidence of malignant brain tumors was 7.08 per 100,000 people, with glioblastoma (GBM, WHO IV high-grade glioma) accounting for around 15% of all CNS tumors (4). Despite advances in surgery, chemotherapy, and radiation, these treatments have not significantly improved the overall survival rates for glioma patients, particularly those with high-grade gliomas such as GBM (5, 6). The blood-brain barrier poses a significant challenge by preventing most antitumor drugs from reaching the brain, thus limiting the effectiveness of systemic therapies (7). Therefore, investigating novel mechanisms and pathway alternations in gliomagenesis may improve diagnostic accuracy, prognosis and overall treatment outcomes for glioma patients.

Glioblastomas are the most malignant and aggressive type of gliomas, characterized with high heterogeneity and mortality. Researchers have showed that gliomas have various subtypes expressing different marker genes including typical NPC-like, OPC-like, AC-like and MES-like profiles using multiple datasets (8). Stress management is critical for cancer cell survival and evolution. Glioma cells generate more reactive oxygen species (ROS) than normal cells due to oncogenic activation, increased metabolic activity and mitochondrial dysfunction, driving heterogeneity. On the other hand, cancer cells may adapt to oxidative stress through mechanisms such as boosting antioxidant status to promotes ROS-driven proliferation while avoiding ROS levels that would cause senescence, apoptosis, or ferroptosis (9–13). Such adaptability leads to malignant transformation, metastasis, and resistance to anticancer treatments (14–17). It is crucial to examine how glioma cells adapt to various stress during the heterogeneity process.

Glioma development and heterogeneity is characterized by gene deregulation through genetic and epigenetic events, as well as cellular damage caused by ROS production (18). Glioma cells maintain metabolic balance by altering the expression of key genes via redox homeostasis pathways such as mitochondrial respiration, stabilizing pro-apoptotic proteins for survival, and preventing apoptosis when challenged. For example, in GBM stem cells (GSCs), activation of AMPK (AMP-activated protein kinase), a master regulator of metabolism, increases glioblastoma bioenergetics and tumor development (19). Another study showed that mitochondrial PKM2 (Pyruvate Kinase M2) regulates oxidative stress-induced apoptosis by stabilizing Bcl2 pro-apoptotic protein (20). Furthermore, the stemness of glioma stem cells is maintained through the interaction of TRAP1 and Sirtuin-3, which modulates mitochondrial respiration and oxidative stress (21). Studies have also found that PTPN2 induced by the inflammatory response and oxidative stress contributed to glioma progression (22). JNK activation increases intracellular ROS production, leading to oxidative stress-induced glioma cell parthanatos (23). Oncostatin M (OSMR) increases mitochondrial respiration by interacting with complex I’s NADH ubiquinone oxidoreductase 1/2 (NDUFS1/2). OSMR deletion reduces spare respiratory capacity, increases ROS, and makes glioma stem cells more susceptible to IR-induced cell death (24). 2-Hydroxyglutarate inhibits transaminase, impairing glutamate biosynthesis and redox homeostasis in glioma (25). Heat shock proteins also protect glioma cells from apoptosis through the endoplasmic reticulum (ER) stress and unfolded protein response pathways. For instance, the Hsp70 chaperone rescues C6 rat glioblastoma cells from oxidative stress by sequestering aggregating GAPDH (26). Recent single-cell RNA-sequencing (scRNA-seq) studies have explored the heterogeneity of gliomas. highlighting the importance of stress responses in heterogeneity and subsequent malignant transformation (27, 28). However, a comprehensive understanding of oxidative stress and its consequences across various gliomas subtypes, particularly at the whole-tissue level, is lacking.

Recent advances in scRNA-seq and spatial transcriptomics (ST) provided ways to visualize and study gene expression in tissue slices, enabling the exploration of transcriptional activity at the single-cell or spatial level (29, 30). scRNA-seq studies have revealed the cellular plasticity and environmental stress response in gliomas (31, 32). Additionally, scRNA-seq has been used to develop comprehensive prognostic signature for glioblastoma patients (33). ST has been applied to various of cancers, including prostate cancer (34), breast cancer (35), and pancreatic ductal adenocarcinomas (36). Our study combines scRNA-seq and ST data from four primary glioma tissue samples to evaluate oxidative stress and its effects. This study aims to elucidate regional and transcriptome-wide expression patterns, enabling that will enable us to deconvolve molecular processes in the oxidative stress response and pathway switching.

## Materials and methods

### Patient recruitment and sample collections

Patients were recruited at the Sichuan Provincial People’s Hospital Neurosurgery Clinic from July 2019 to September 2020. Detailed verbal and written information about the study was provided to all participants, who gave written informed consent before participating. from the study included two untreated patients: one female with WHO grade IV glioblastoma in the left temporal lobe (high-grade glioma, HG) and one male with WHO grade II oligodendrocyte astrocytoma in the right temporal lobe (low-grade glioma, LG). Detailed basic clinical and pathological information for the participants is provided in **Supplementary Table 1**.

### Single-cell transcriptomic profiling of glioma samples

Four fresh samples were collected prior to surgery and processed into single-cell suspensions. These samples were analyzed using droplet-based single-cell RNA sequencing (scRNA-seq) technology of the 10 x Genomics Chromium system. The Cell Ranger software (version 3.1.0) was employed for initial data processing, including alignment and quality control. Post-quality control, the number of high-quality cells in each sample ranged from 2,419 to 10,733. Low-quality cells (doublets, multiplets, and apoptotic cells) were excluded, resulting in final cell counts ranging from 1,606 to 9,744. The average number of Unique molecular identifiers (UMIs) per cell ranged from 7,325 to 15,424, the average number of genes per cell is 2,424 to 3,858, and the mitochondrial genes ratio per cell from 5.69% to 11.90%. We used the R package Seurat (version 3.1.1) to process the filtered unique molecular identifier (UMI) count matrix (33), with variable gene identification using the algorithm provided by Macosko et al. (37) Principal component analysis (PCA) was used in Seurat to reduce dimensionality using the RunPCA function (PC num = 15) (33). Clusters were analyzed using FindClusters function and visualized via t-distributed stochastic neighbor embedding (t-SNE) (RunTSNE). Marker genes for each cluster were identified using the FindAllMarkers function in Seurat (33). Then, cell types were inferred using the R package SingleR, an automated annotation method for unbiased scRNA-seq cell type detection, with Human Primary Cell Atlas from Mabbott et al. as the reference transcriptome datasets, to infer the cell types (38, 39). Differentially expressed genes (DEGs) were identified with FindMarkers function in Seurat. The criterion for substantially different expression was established at *P* Value < 0.05 and |log_2_foldchange| > 0.58. Gene Ontology (GO) and KEGG Kyoto Encyclopedia of Genes and Genomes (KEGG) pathway enrichment analyses were performed on of DEGs using hypergeometric distribution.

### 10x Visium spatial RNA-seq data preprocessing

Spatial transcriptomic analysis was conducted using the 10x Genomics Visium platform. The Space Ranger software (version 1.0.0) was used for tissue identification, read alignment via the STAR aligner, feature-spot matrix construction, clustering, gene expression analysis, and spatial visualization slide images (40). This analysis contains the spatial transcriptome sequencing of 4 samples with 4 slices. The number of high-quality spots for Space Ranger quantitative quality control of each sample ranges from 2,791 to 3,821. The average UMI counts per spot ranges from 7,113 to 22,143, and the average number of genes per spot from 3,093 to 6,002, and mitochondrial genes ratios per spot ranges from 8.24% to 13.21%. To process the filtered unique molecular identifier (UMI) count matrix, the R package Seurat (version 3.1.1) was utilized (33). We initially standardized the data to account for differences in sequencing depth across data points before identifying high-variance features and storing the data in the SCT assay (41). FindClusters function was applied to analyze cell groups based on their gene expression profiles using graph-based clustering in Seurat. We used the RunTSNE function to display clusters using a 2-dimensional t-distributed stochastic neighbor embedding (t-SNE) method and the FindAllMarkers function to find marker genes in each cluster (33).

### Copy number variation analysis

Genomic copy number and subcolonal structure of human tumors were predicted using the CopyKat and Infercnv packages, enabling high-throughput genomic analysis from scRNA-seq data (42–44).

### Multimodal intersection analysis

Multimodal intersection analysis (MIA) was used to annotate spatial transcriptome spot clusters with single-cell transcriptome data, enhancing cell type resolution at the spot cluster level.

### Histopathological examination

The collected glioma tissues were paraffin-embedded, sectioned, and stained with hematoxylin and eosin (H&E) for morphological analysis. For immunohistochemistry (IHC), the sections were routinely incubated with the c-Jun antibody (10586-1-AP from Proteintech, 1:200), and processed using a rabbit IgG-immunohistochemical SABC kit (Boster, Wuhan, China). After PBS washing, sections were counterstained with hematoxylin for nuclei staining and examined under an Olympus microscope (Japan).

## Results

### ScRNA-seg results reveal the links between heterogeneity and malignancy in gliomas

**Figure 1:**
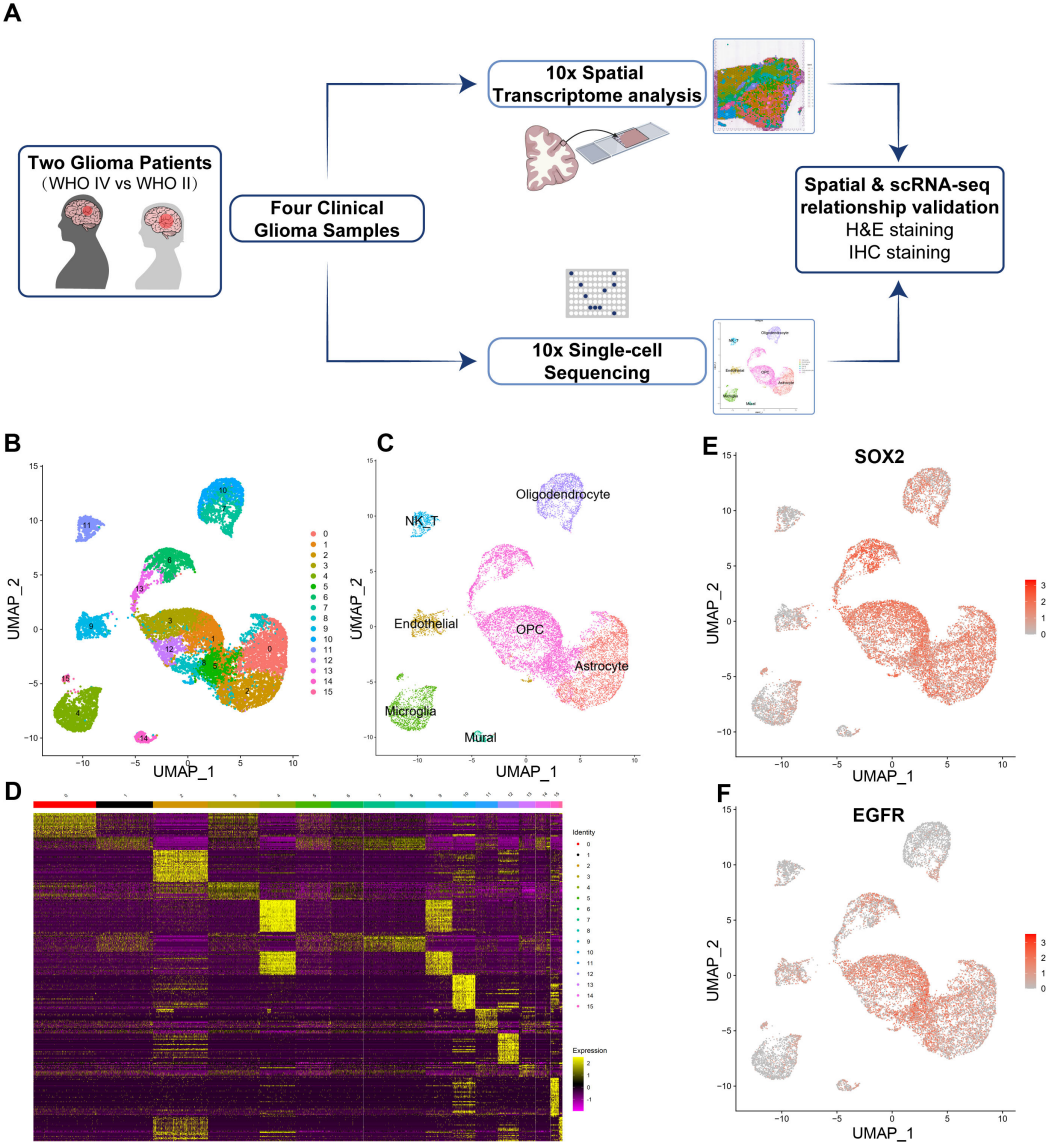
Isolation and cluster analysis of single-cell transcriptomes from four glioma samples derived from two patients. **(A)** Workflow of experimental strategy: isolation of human glioma tissues during clinical surgery, followed by parallel scRNA-seq and Spatial Transcriptomic (ST) analysis profiling using with the 10x Genomics Chromium platform and subsequent validation by H&E and IHC staining. **(B)** Uniform Manifold Approximation and Projection (UMAP) plot showing 16 major clusters. **(C)** Clusters are annotated for their cell types as predicted using canonical markers and signature-based annotation using Garnett. **(D)** Heatmap showing clustering with top 30 highly expressed genes. **(E, F)** Feature UMAP plots depicting cluster-specific expression of cell clusters markers including SOX2 (SRY-Box Transcription Factor 2) and EGFR (Epidermal growth factor receptor) to indicate the major malignant cell clusters.

To explore the cell compositions in glioma tissues, scRNA-seq was conducted on all living cells isolated from four fresh primary glioma samples from two untreated patients with the 10x Genomics Chromium platform for parallel scRNA-seq and ST analysis (**Figure 1A** and method). The scRNA-seq data consisted of cells with approximately 7,325–15,424 unique molecular identifiers (UMIs) and approximately 2,424–3,858 uniquely expressed genes per cell (**Supplementary Figure 1**). The average percent of mitochondrial, ribosome, and hemoglobin subunit genes were also calculated and used for further data normalization and batch effects between two samples removed using harmony package (45) (**Supplementary Figure 1**). We found 16 cell clusters in this integrated scRNA-seq data (**Figure 1B**). We identified them as endothelial cells (ECs), natural killer T cells (NKTs), mural cells, microglia, oligodendrocytes, and malignant cells utilizing cell-type-specific marker genes (**Figure 1C**). The visualization plot was generated using the dimensionality reduction method UMAP (uniform manifold approximation and projection). The heatmap of the top 30 genes from each cluster is shown in **Figure 1D**. Moreover, the tSNE (t-distributed Stochastic Neighbor Embedding plot) for the expression of marker genes from each cell type is shown in **Supplementary Figure 2A**. Two immune-cell clusters (clusters 4, 11) were initially identified based on the expression level of *PTPRC* (CD45), a pan leukocyte marker (**Supplementary Figures 2B**; **3A**). According to the presence of the typical marker *CSF1R* (Colony Stimulating Factor 1 Receptor, CD115), cluster 4 was mononuclear phagocytes (microglia) (**Supplementary Figure 2D**). Cluster 11 contains Natural Killer/T cells (NK/T), which express typical markers such as *CD8A*, *CD8B*, *NKG7*, *CCL4*, *CCL5*, but not *CCR7*, *CD4*, *CTLA4*, *FOXP3*, and *IL2RA* (**Supplementary Figures 2C**; **3A**). In our glioma samples, this observation suggested the existence of CD8+ T cells rather than naive or CD4+ T cells. Cluster 9 has been identified as endothelial cells expressing specific markers such as *FLT1*, *ICAM1*, *CDH5*, *CD34*, and *ENG* (CD105) (**Supplementary Figures 2E**; **3B**). Mural cells, as an integral part of the neurovascular unit, make up the majority of cluster 16, expressing specific markers such as *ACTA2*, *PDGFRB*, *ZIC1*, *ABCC9*, *RGS5*, *CSPG4*, and *MCAM* (CD146) (**Supplementary Figures 2F**; **3C**). Clusters 7 and 8 are mature oligodendrocytes that produce myelinating oligodendrocyte markers, including *SOX10*, *CNP*, *MAG*, *MOG*, *MBP*, and others (**Supplementary Figures 2J**; **3D**). However, they do not express proliferative factors such as *EGFR*, *PDGFRA*, or *L1CAM*. Cluster 1 and 12 expresses GFAP, an astrocyte marker, and are therefore classified as astrocytes. All other clusters express *OLIG2*, *OLIG2*, *PDGFRA*, and *NG2* (CSPG4), indicating that they are OPC groups. In another cell cluster, we also discovered the high expression of well-known cancer stem cell markers *SOX2* (**Figure 1E**) and *EGFR* (Epidermal Growth Factor Receptor), indicating abnormal proliferation and malignancy in these cell clusters (**Figure 1F**).

### Malignant cell heterogeneity and copy number variation analysis

Previous research suggested that four cellular states, NPC-like, OPC-like, AC-like, and MES-like, drive glioblastoma malignant cell heterogeneity. These malignant cells were identified OPC-like cell expression markers like *OLIG*1 *OLIG1*, *OLIG2*, *PDGFRA*, and *NG2* (CSPG4), indicating that they are OPC groups. Infercnv package in R to further validate the malignant cells to calculate the copy number variations (CNVs) (42, 46) (**Supplementary Figure 5**). The malignant cell group showed a great heterogeneity. As we expected, the OPC-likes have the most significant alternation in CNV levels (**Supplementary Figures 5B–D**). According to the heatmap, we observed amplification of CNV of chromosome 7/10, a molecular marker of glioma, while a loss of chromosome 11 from the predicted results (**Supplementary Figure 5A**).

### Spatial transcriptomics reveals distinct tumor microenvironments in high-grade and low-grade gliomas

**Figure 2:**
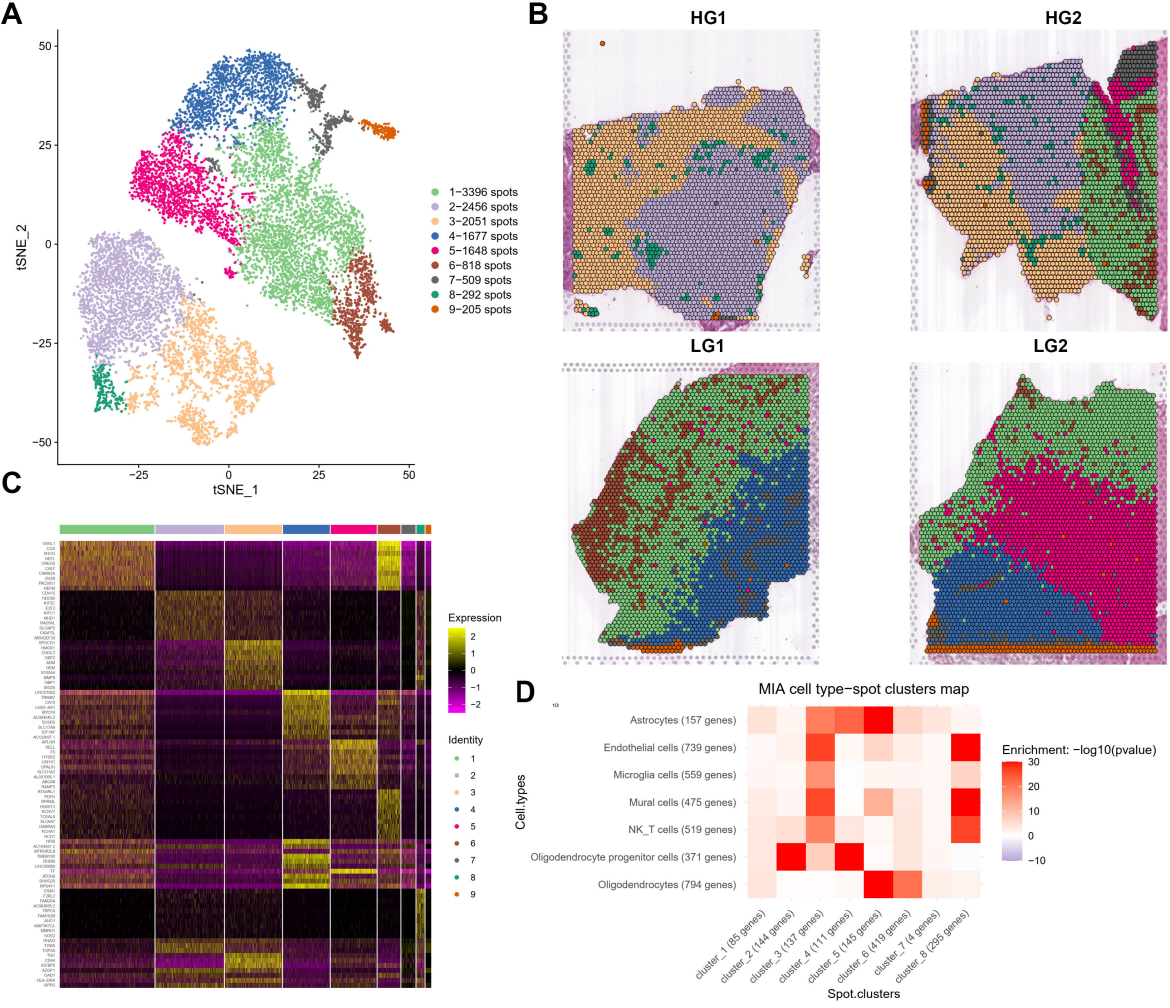
Cluster analysis and annotations of spatial transcriptomes from four glioma samples derived from two patients. **(A)** t-distributed stochastic neighbor embedding (tSNE) projection of spots showing 9 major ST clusters. **(B)** The spatial relationship among 9 major ST clusters in four different samples. **(C)** Heatmap showing clustering with top 30 highly expressed genes from each ST clusters. **(D)** Heatmap showing the correlation matrix generated from 8 major ST Clusters and 7 major cell types from scRNA-seq calculated through MIA (Multimodal intersection analysis).

For ST data, we performed the integrated clustering analysis of ST and revealed 9 different spatial clusters (**Figure 2A**). A significant variation in spatial spot type was observed between HG and LG to explain the heterogeneity. The spatial characteristics and cluster distribution of HG and LG are distinct (**Figure 2B**). Spatial cluster1 and cluster5 are primarily spread on the HG2 edge and the core portions of LG1 and LG2. While spatial cluster 1 was considered as mixed cell types, spatial cluster 5 was labeled as oligodendrocytes due to its MAG, MOG, GFAP, and MBP expressions. It was mostly found in LG2, although it was also discovered in the HG2 marginal region. Spatial cluster 8 was found in both HG1 and HG2, and it was apparently dispersed around the core tumor region (**Figure 2B**). The heatmap for the top 30 genes from each ST cluster was generated (**Figure 2C**). Interestingly, the extracellular matrix protein-encoding genes such as *FN1*, *TIMP1*, *COL3A1*, *COL4A1*, and *COL2A1* were discovered in spatial cluster 8, regarded as tumor stroma tissues. Endothelial markers such as *PECAM1*, *CD93*, and *ENG* were also strongly expressed in spatial cluster 8. Cluster 4 was considered tumor stroma tissues mainly represented by fibroblasts. The correlations between ST clusters and scRNA-seq cell types were shown by integrative analysis of cell data and spatial transcriptome data using Multimodal intersection analysis (MIA). As shown in the heatmap of **Figure 2D**, clusters 2 and 4 are most likely to be malignant OPC-likes, while clusters 5 and 6 could be oligodendrocytes or astrocytes. Clusters 3 and 8 might be a mixture of immune cells, endothelial cells, and mural cells (**Figure 2D**).

We next annotated the ST spot using SingleR and examined each sample individually to validate the results, considering tumor heterogeneity and individual differences among patients. The most frequent types of malignant cells in HG gliomas are MES-like (orange), OPC-like (yellow), and unidentified malignant type (grass green) (**Supplementary Figures 4A, B**). Oligodendrocytes (red) and AC-like malignant cells (dark blue) were found near the periphery of HG2, which can be differentiated from the primary malignant cells in HG1 (**Supplementary Figure 4B**). Normal immune cell infiltration also differs significantly between HG and LG gliomas. For example, monocyte infiltration (light purple) is more clearly observed in HG glioma tissue (HG1 and HG2), likely due to the looser structure of high-grade tumors. In contrast, LG gliomas (LG1 and LG2) have a tighter arrangement of tumor cells, and limiting immune cell infiltration. The great majority of malignant cells in LG glioma spatial spots are categorized as OPC-like (yellow), unidentified (grass green), or AC-like (dark blue), with NPC-like (purple) and MES-like (orange) malignant cells being uncommon (**Supplementary Figure 4C**). OPC-like-malignant is primarily found on the interior of the sample, whereas AC-like-malignant is more prevalent on the exterior. Simultaneously, LG2 contains more OPC-like-malignant components, while LG1 has more AC-like-malignant components (**Supplementary Figure 4D**).

### OPC clusters revealed activation of stress response genes

**Figure 3:**
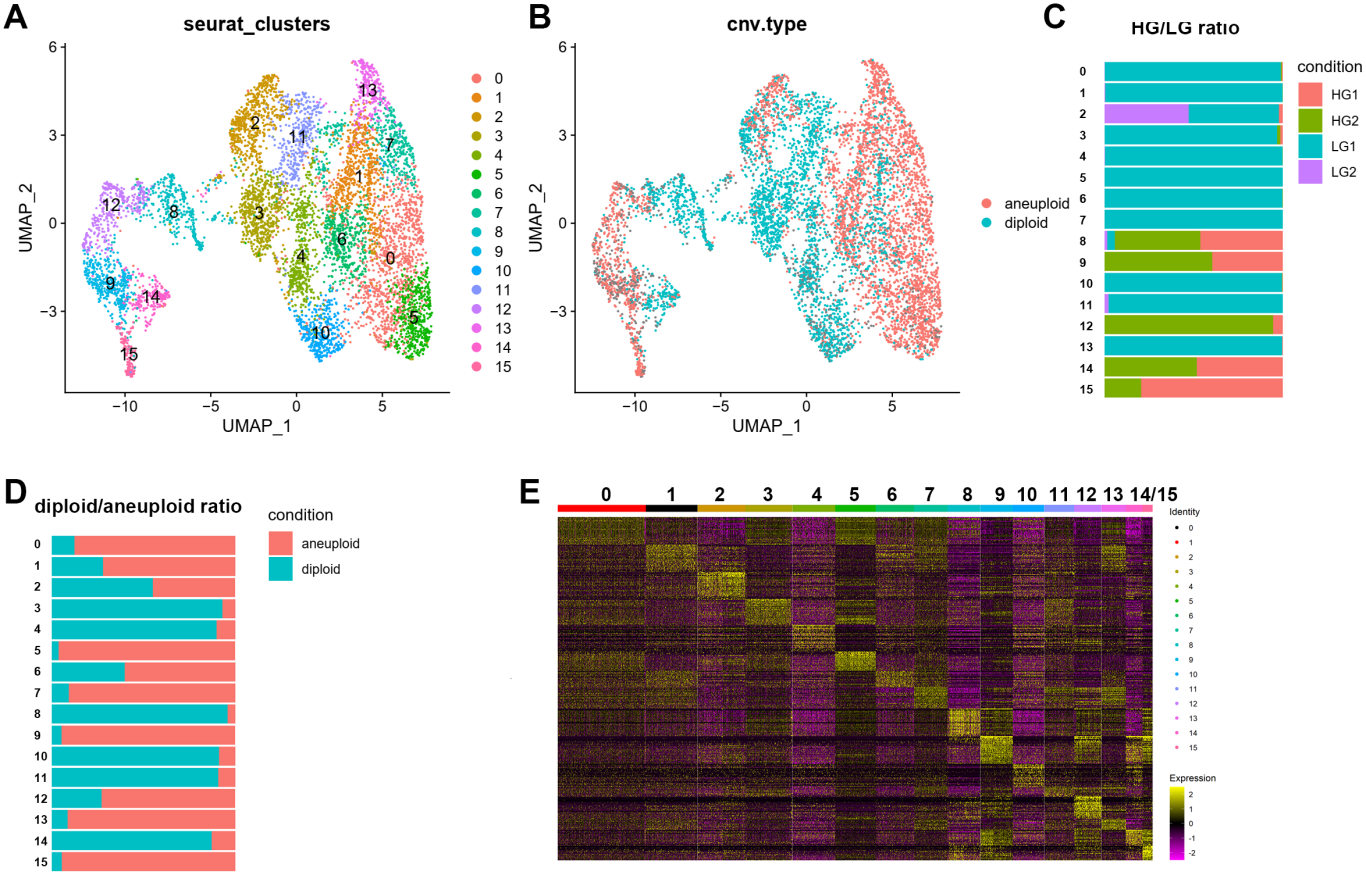
Single-cell RNA-seq of OPC cell populations. The OPC subclusters were extracted from the whole scRNA-seq data and processed for dimensionality reduction. **(A)** Uniform Manifold Approximation and Projection (UMAP) plot showing 16 major clusters of 7221 OPC single cells. **(B-D)** UMAP plot with CNV (copy number variation) annotations according to CopyKat package calculations. Aneuploid was depicted as red while diploid was shown as blue. Stacked bar charts showing the distribution of 16 subclusters of OPC with its sample origin and predicted CNV status. **(E)** Heatmap showing clustering with top 30 highly expressed genes from each OPC subclusters.

We performed a PCA dimensionality reduction analysis on the OPC subpopulation single-cell data and used UMAP projection to obtain the expression matrix (**Figure 3A**). This analysis revealed 16 subclusters of OPC-like cells (**Figure 3A**). Using the CopyKat package, we assessed malignant and normal cells based on predicted aneuploid and diploid states to detect aberrant cells in single-cell clusters. The copy number variation (CNV) data show differences in OPC-like subclusters, with normal diploid cells primarily identified in subclusters 3, 4, 8, 10, 11, and aneuploid cells mainly discovered in other subclusters (**Figures 3B, C**). Both LG and HG samples shared Clusters 3 and 8 (**Figure 3D**). A heatmap displaying the top 30 genes from each OPC-like subcluster is shown in **Figure 3E**.

**Figure 4:**
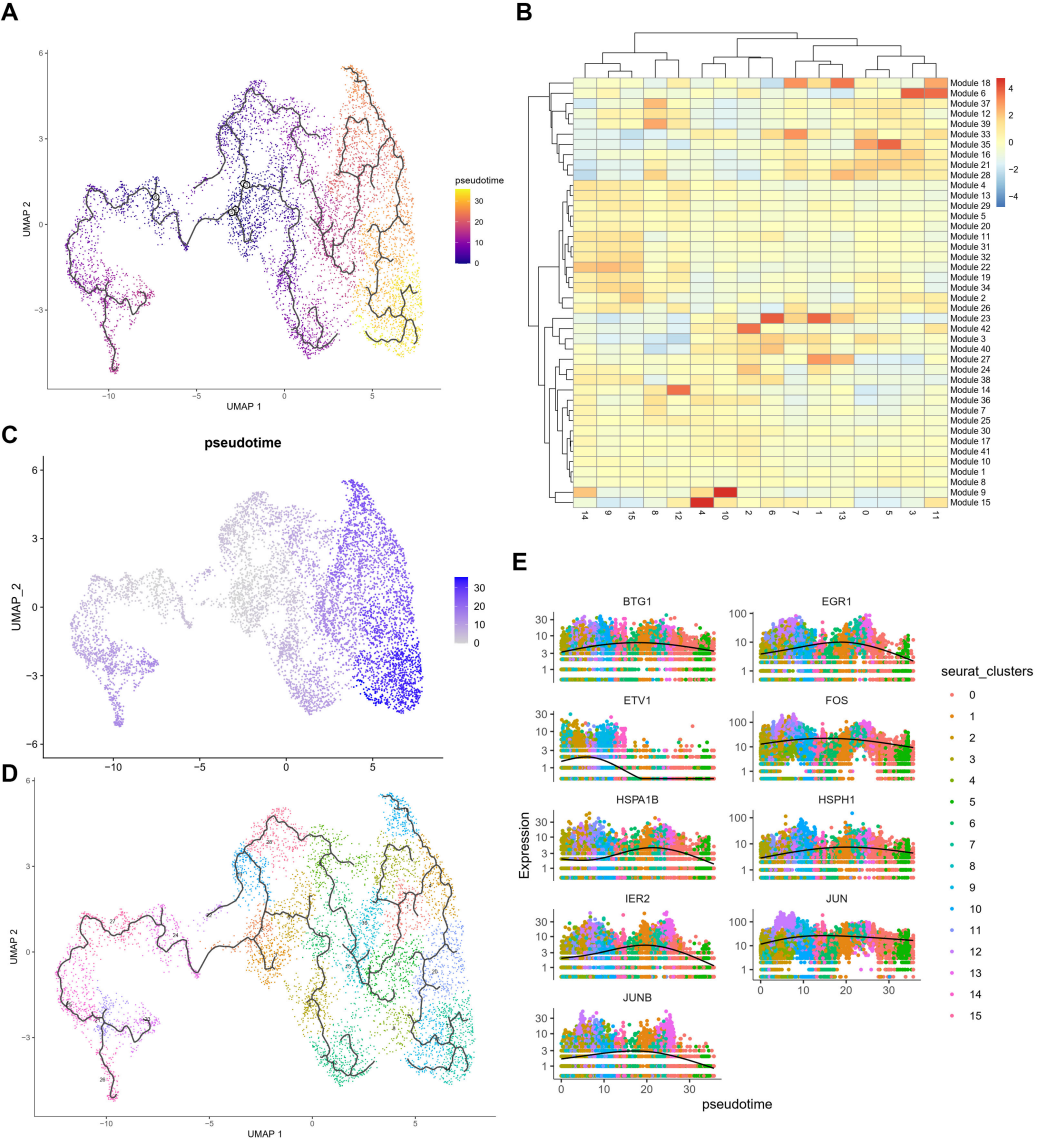
Pseudo-time analysis of OPC subclusters using Monocle3 **(A)** Monocle3 was used to track cells over pseudotime on the different OPC subclusters. **(B)** The heatmap of 39 modules calculating the gene expressions after prediction of trajectory. **(C, D)** Visualization of clusters (from **Figure 1C**) onto the pseudotime map. **(E)** Pseudo-time plots of gene expression for stress response genes (*BTG1*, *EGR1*, *ETV1*, *FOS*, *HSPA1B*, *HSPH1*, *IER2*, *JUN* and *JUNB*) with cells colored by OPC subcluster group indicating the similar patterns among those genes.

Using Monocle3, we set the start points of the pseudotime intervals to OPC-like subclusters 3 and 8 (**Figure 4A**). According to Monocle3 data, three primary lineages emerge from the starting point (**Figures 4C, D**). We created 28 distinct modules with varying gene expression patterns (**Figure 4B**) and found that several stress response genes exhibit distinct expression patterns. Oxidative stress-related genes with specific expression patterns include BTG1, EGR1, ETV1, FOS, HSPA1B, IER2, JUNB, JUN, DNAJB1, and HSPH1, *all* showing very similar trajectories (**Figure 4E**). **Supplementary Figure 6** demonstrates the elevated levels of oxidative stress response genes and the elevation in pseudotime in an OPC-like subcluster UMAP plot, including stress response transcription regulators (BTG1, EGR1, FOS, IER2, JUNB, JUN) (**Supplementary Figure 6A**) and heat shock proteins (HSPH1, HSPA1B), as well as DNA damage response gene DNAJB1 (**Supplementary Figure 6B**). Additionally, tumor proliferation regulator and metabolic genes implicated in glycolysis and gluconeogenesis pathways, including VEGFA, CCN1, VIM, LDHA, LDHB, PKM, ENO1, and PGK1, were up-regulated in HG OPC-like subclusters (data not shown), indicating an increase in stress response during the gliomagenesis.

### Specific expression patterns of stress response genes near the necrotic core

We validated the expression and cell-type distribution of these stress response transcription factors (TFs) using the Ivy Glioblastoma Atlas Project (**Supplementary Figure 7**). The peri-necrotic zone and pseudo-palisading cells around necrosis showed significantly higher mRNA levels of JUN, JUNB, FOS, and FOSL2 compared to other regions of the tumor, such as the leading-edge, infiltrating tumor, and cellular tumor (**Supplementary Figures 7A-D**). Pseudo-palisading cells around central degeneration have been recognized for nearly a century as a hallmark of GBM and indicate aggressive tumor behavior. The enrichment of FOSL2 protein in the peri-necrotic zone and pseudopalisading cells area was further confirmed by in situ hybridization (ISH) and H&E staining with tumor feature annotations (**Supplementary Figures 7E, F**).

Additionally, H&E staining of primary glioblastoma tissues from three patients (A020-21621, A021-12863, A021-15304) during surgery illustrated the representative necrotic areas and pseudo-palisading regions (**Supplementary Figure 8A**). The elevation of JUN protein expression around these areas was confirmed by immunohistochemical staining (IHC) using the specific antibody against JUN (**Supplementary Figure 8B**). Data from GEPIA2 also showed frequently increased FOS, JUNB, JUN, and ATF3 in glioma, especially GBM samples, compared to normal brain tissues (**Supplementary Figure 9**). These findings suggest that JUN family members are important regulators in glioma stress response, especially in GBM.

### Oxidative stress response in high-grade glioma

**Figure 5:**
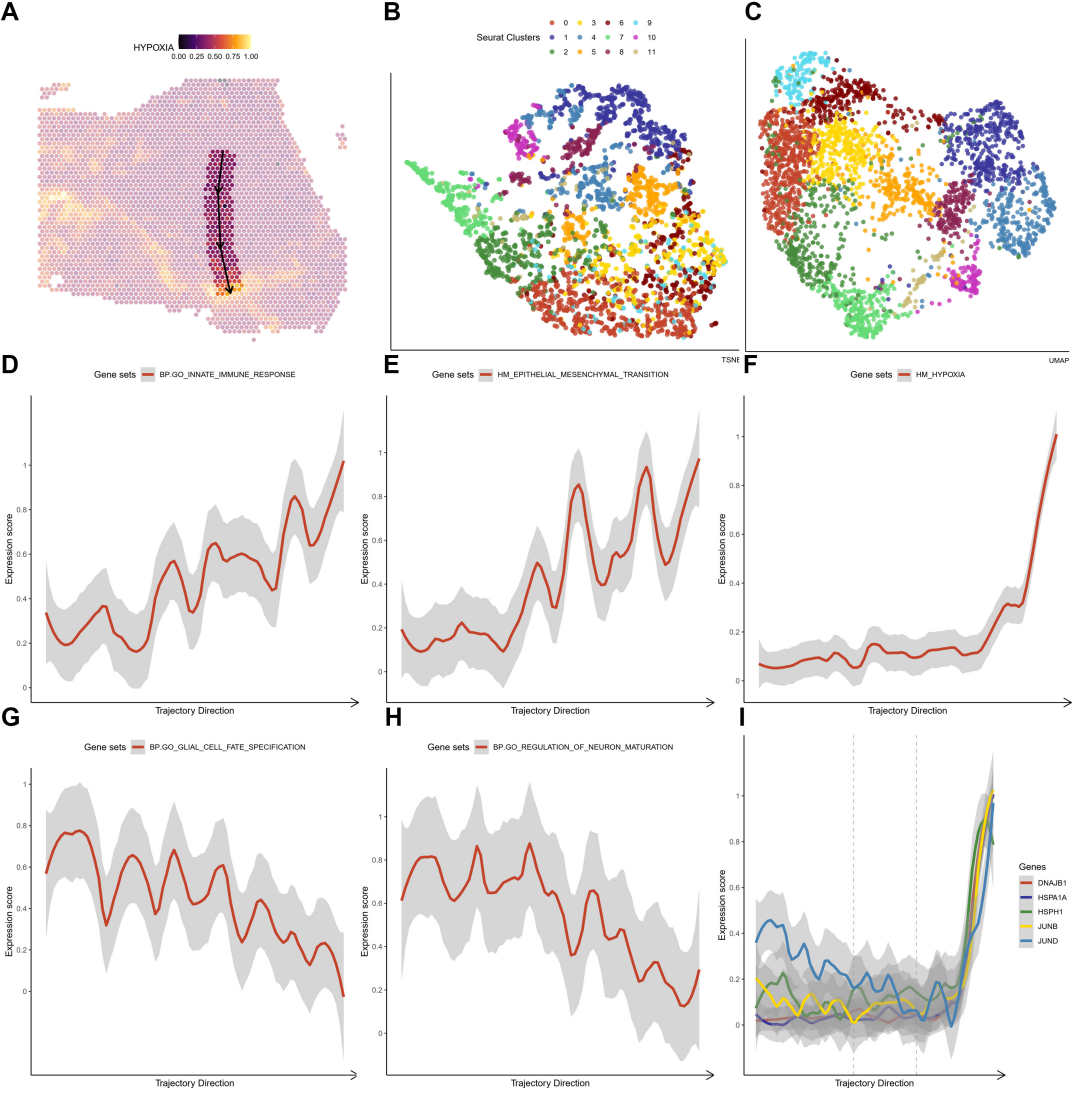
The crucial pathway altered in the trajectory under an increasing hypoxia stress in HG1 **(A)** The trajectory in the HG1 with a increasing hypoxia score. **(B, C)** tSNE and UMAP plot showing distribution of different ST clusters. **(D-H)** The significantly altered pathways either descending or ascending in the trajectory. **(I)** The expressions of marker genes (DNAJB1, HSPA1A, HSPH1, JUNB and JUND) in the trajectory that response to hypoxia stress.

**Figure 6:**
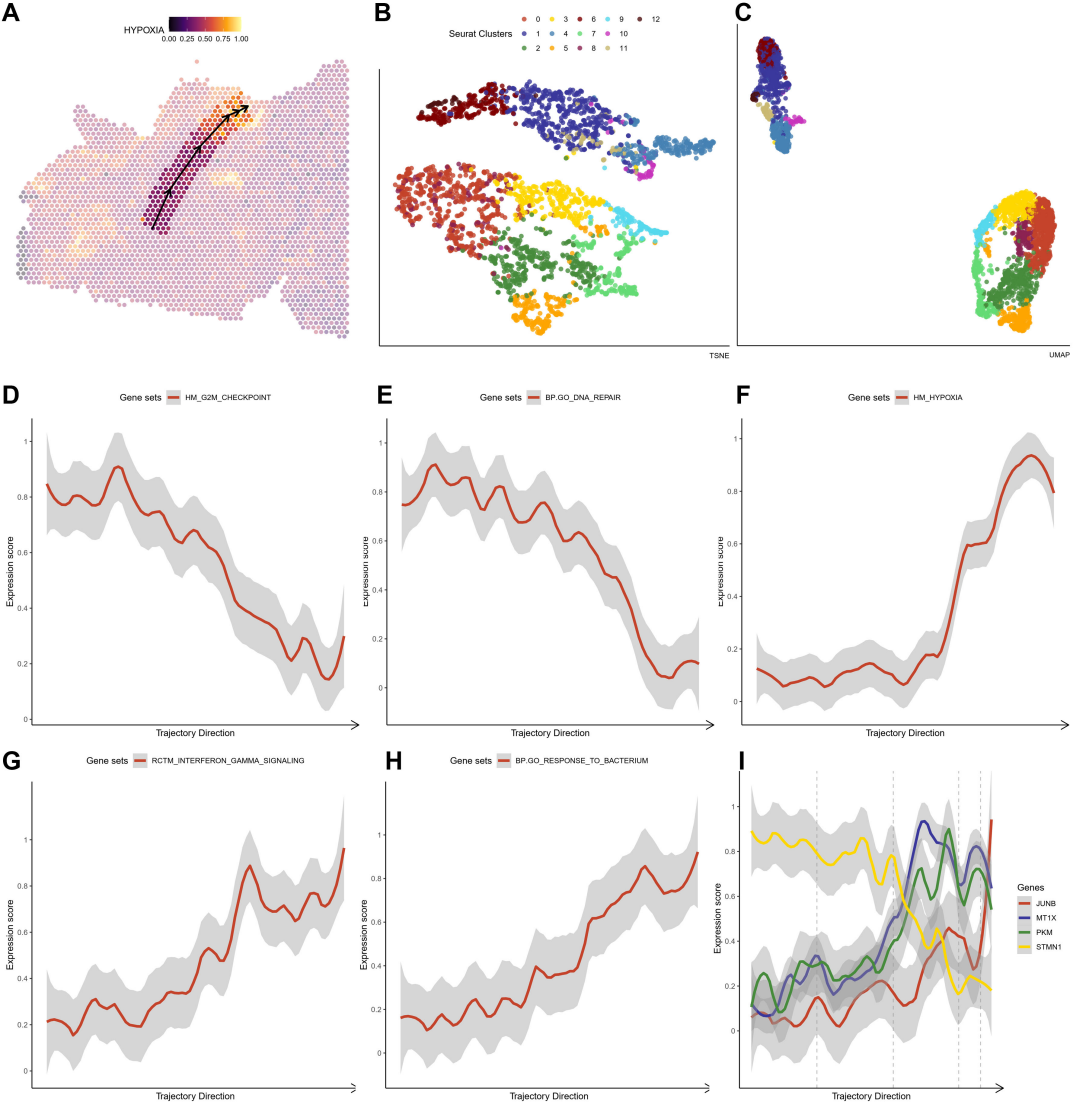
The crucial pathway altered in the trajectory under an increasing hypoxia stress in HG2 **(A)** The trajectory in the HG2 with an increasing hypoxia score. **(B, C)** tSNE and UMAP plot showing distribution of different ST clusters. **(D-H)** The significantly altered pathways either descending or ascending in the trajectory. **(I)** The expressions of marker genes (*JUN, MT1X, PKM* and *STMN2*) in the trajectory that response to hypoxia stress.

For both HG1 and HG2, we used the ST transcriptome data and an enrichment study in R studio using the SPATA package (version 1.0.1). We discovered that the hypoxia response (Hall-Mark hypoxia pathway) is enriched in twisted bands or spots around the central areas (**Figures 5A**; **6A**). Following that, we utilized the Monocle3 package (version 1.0.1) to determine the trajectory of gene alterations in the process of transitioning from a low hypoxic response to a high hypoxic response in both HG1 and HG2. Dimensionality reduction plots indicate cell population clustering in UMAP and t-SNE for HG1 and HG2 (**Figures 5B, C**; **6B, C**).

As predicted, the hypoxia response increases substantially throughout this phase (**Figure 5F**). In HG1, increasing routes include the initial immune response pathway (BP: GO) (**Figure 5D**) and the epithelial-mesenchymal transition (Hallmark) (**Figure 5E**), while decreasing pathways include glial cell differentiation (BP: GO) (**Figure 5G**) and nerve cell maturation (BP: GO) (**Figure 5H**). This suggests that the tumor cells advanced from a somewhat benign condition to a dedifferentiated or even invasive status in the central high-grade tumor sample. The rapid elevation of oxidative stress response gene expression, including JUNB, JUN, DNAJB1, HSPA1A, and HSPH1, was shown under oxidative stress (**Figure 5I**). On the other hand, hypoxia has a greater impact on cell cycle regulation (**Figures 6D, F**), DNA replication (**Figures 6E, F**), and cell proliferation in HG2. We also found that immune responses, such as the response to bacteria (**Figures 6F, G**) and interferon-gamma signaling (**Figures 6F, H**), are highly activated in the hypoxia regions in HG2, located in the marginal areas of high-grade glioma. This might be triggered by external stimuli in the brain.

**Figure 7:**
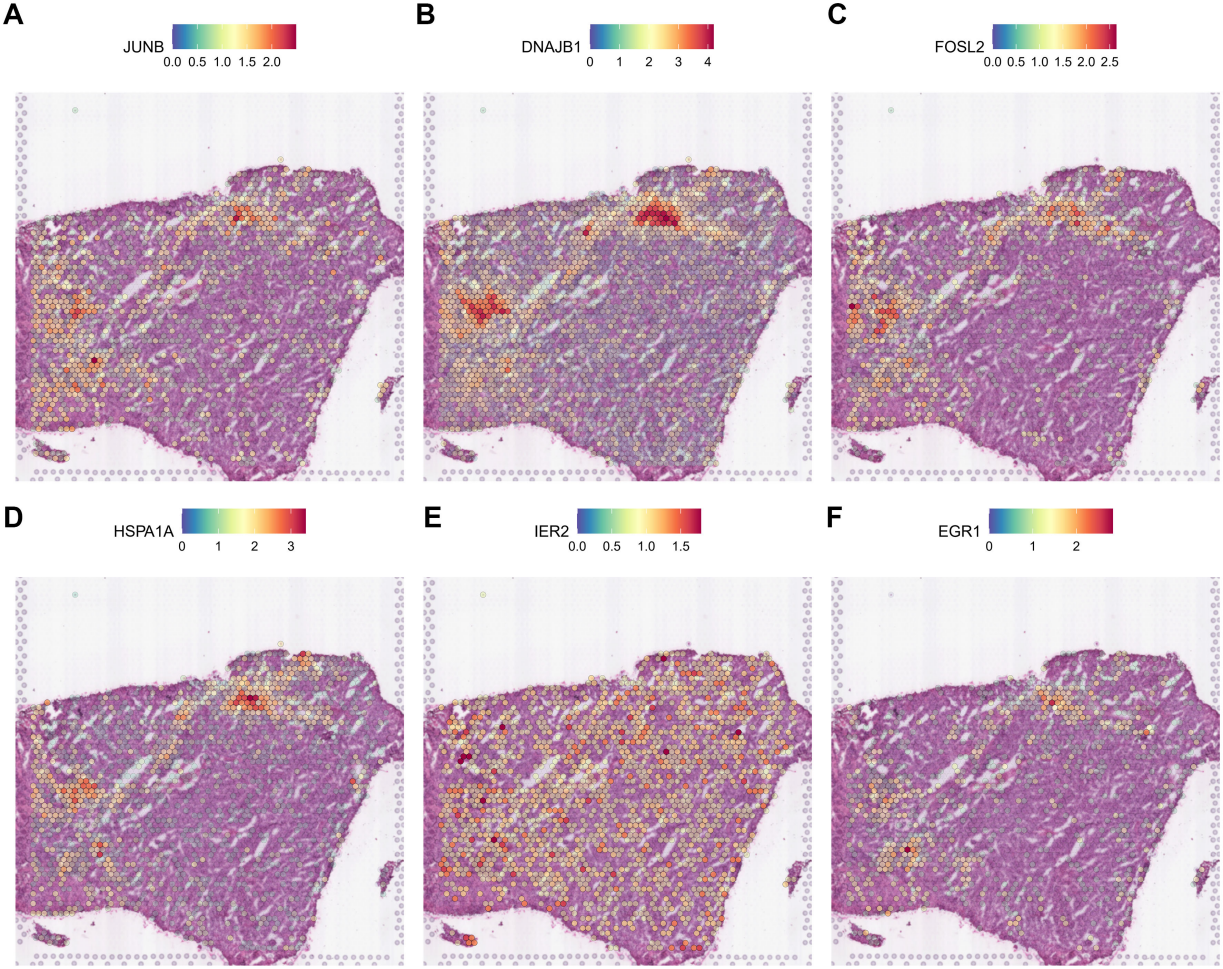
Expression patterns of stress response genes in HG1 spatial transcriptomics data. **(A-F)** The expressions of stress response gene including *JUN, DNAJB1, FOSL2, HSPA1A, IER2* and *EGR1* was shown in the ST profile from HG1 tissues.

**Figure 8:**
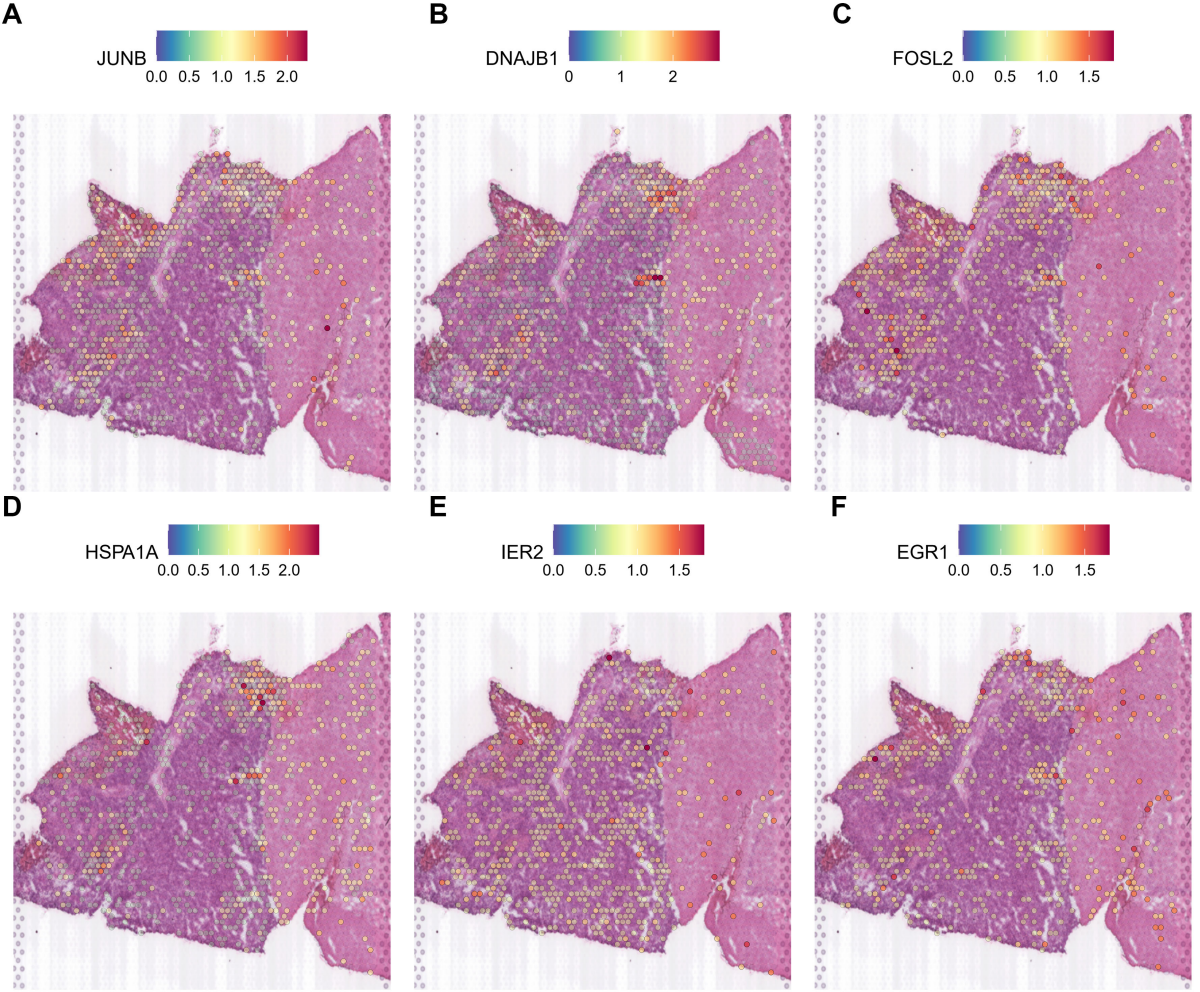
Expression patterns of stress response genes in HG2 spatial transcriptomics data. **(A-F)** The expressions of stress response genes including *JUN, DNAJB1, FOSL2, HSPA1A, IER2* and *EGR1* was shown in the ST profile from HG2 tissues.

Thus, it is clear that during gliomagenesis, cells first undergo uncontrolled proliferation and then respond to stress by abandoning the essential features of glial cells, leading to malignant transformation. Consistent with these findings, we discovered that tumor-proliferative factors such as *VEGFA* (Vascular Endothelial Growth Factor A), the representative downstream regulator of epithelial-mesenchymal transition, *VIM* (Vimentin), and the inflammatory cytokine SPP1, which regulates endoplasmic reticulum stress, are all significantly up-regulated in peripheral hypoxic regions in both HG1 and HG2 samples (**Supplementary Figures 12**, **14**). The subcluster of OPC-like was projected using the ST data; cluster 12 was regarded as malignant, and cluster 3 was less malignant (**Supplementary Figures 13**, **15**). Meanwhile, genes that have functions in the normal brain or neurons, such as *CaMK2N1* (Calcium/Calmodulin Dependent Protein Kinase II Inhibitor 1)*, MEG3* (Maternally Expressed 3), *SNCB* (Synuclein Beta), *SYT1* (Synaptotagmin 1), *DKK3* (Dickkopf WNT Signaling Pathway Inhibitor 3), are almost not expressed in the tumor core. Additionally, stress-response transcription factors (*JUNB, FOSL2, IER2, EGR1*), DNA damage response proteins (*DNAJB1*), and heat shock proteins (*HSPA1A*) are dramatically up-regulated in the hypoxia regions (**Figures 7**, **8**). They are concentrated in certain punctate areas, which overlap with hypoxia response areas in HG1 (**Figure 7**). However, on the other hand, those genes in HG2 are not as active as they are in HG1 regions. While the hypoxia score is growing in the trajectory, there is only a rapid increase in *JUNB* expression in HG2 (**Figure 8**).

### Oxidative stress response in low-grade glioma

We use the Seurat tool to divide the spatial subgroups into 12 groups for both LG1 and LG2. After enrichment analysis by SPATA package in R studio (**Supplementary Figures 10A**; **11A**). We used the Monocle3 package analysis of the SPATA toolkit to establish the trajectory of gene changes in the process from a hypoxic response to a high hypoxic response (from 0.00 to 1.00) (**Supplementary Figures 10D**; **11D**). Unlike high-grade gliomas, the hypoxic response process increases slowly and steadily in low-grade gliomas. They are distributed on the edge of low-grade gliomas in a diffuse state. And the accompanying pathways are mainly normal nerve functions, such as synaptic endocytosis (**Supplementary Figure 10E**), neural projection (**Supplementary Figure 11H**), neurotransmitter secretion (**Supplementary Figure 11E**), and presynaptic activity area. The accompanying pathways are mainly normal nerve functions, such as synaptic endocytosis, neuroprotection, neurotransmitter secretion, and presynaptic activity area. The differentiation of OPC from core areas into the oligodendrocytes triggered the hypoxia areas in LG. Also, increased expression of stress response genes such as *JUNB* and *FOSL2* is observed in the trajectory of LG1 (**Supplementary Figure 11I**) but not in LG2 (not shown). Neuron functional genes such as *CaMK2N1, MEG3*, *SNCB*, *SYT1*, *DKK3* are almost expressed in the marginal area of the tumor (not shown). According to previous MIA analysis, it is believed that it is mainly oligodendrocytes. We believe that the increased oxidative stress is caused by the differentiation of OPC cells in glioma.

### The metabolic shift in different types of glioma spatial subtypes

Using spatial transcriptome data, we uncovered an unexpected fact that mitochondrial gene expression differs in high grades of gliomas. In HG1, we found that all 13 human mitochondrial genes in HG1 were primarily expressed in normal oligodendrocytes or AC-like malignant cells rather than OPC-like or MES-like malignant cells (*MT-ND1*, *MT-ND2*, *MT-ND3*, *MT-ND4L*, *MT-ND4*, *MT-ND5*, *MT-ND6*, *MT-CYB*, *MT-CO1*, *MT-CO3*, *MT-ATP6*, *MT-ATP8*, *MT-RNR2*) except for *MT-CO2* (**Supplementary Figure 20**). In the tumorous sections of HG2, the mitochondrial genes *MT-ND1*, *MT-ND2*, *MT-ND4*, *MT-ATP6*, *MT-CYB*, *MT-CO3* were completely absent as compared to the normal parts, as shown in **Supplementary Figure 21**. A similar situation also occurred in HG1; the hypoxic area of HG1 nearly overlaps with the regions that have a poor expression of mitochondrial genes such as *MT-CO3*, *MT-ATP6*, *MT-CO1*, *MT-ND3*, *MT-CYB*, and *MT-ND1*. This finding suggests that mitochondrial dysfunction might be a hypoxia-induced cellular disorder. However, the results will need to be double-checked in further study. We saw a fast elevation in glycolysis-related genes such as *PGK1* (Phosphoglycerate Kinase 1), LDHA (Lactate Dehydrogenase A), *PKM2* (Pyruvate Kinase M2), and *ENO1* (Enolase 1) in both HG1 and HG2 at the same time that mitochondrial genes were absent. When taken together, these findings imply that the tumor metabolic model might have changed under hypoxia stress, possibly shifting from mediated by mitochondria to glycolysis or gluconeogenesis. In The Cancer Genome Atlas (TCGA)-GBM/low grade glioma (LGG) (**Supplementary Figure 16**) and CGGA datasets (**Supplementary Figure 18**), we observed a decrease of MT genes in both datasets. Interestingly, mitochondrial gene expression is related to the overall survival of patients with glioma in the CGGA and TCGA-GBM/LGG datasets (**Supplementary Figures 17**, **19**). The percentage of mitochondrial genes was decreased in HG1 and HG2 samples; however, the nUMI and nGene were increased (**Supplementary Figure 22**). These results indicated a stress response alternation in metabolism for HGs. The metabolic shift allows glioma cells to generate energy more rapidly and supports biosynthetic processes essential for rapid growth. By relying on glycolysis, glioma cells reduce oxidative stress and ROS production, which helps them evade apoptosis and resist cell death. Additionally, this metabolic reprogramming aids in immune evasion and correlates with increased tumor aggressiveness, contributing to a poorer prognosis for patients with high-grade gliomas (47, 48). In summary, this metabolic shift from oxidative phosphorylation to glycolysis is crucial for glioma progression as it enhances tumor proliferation, survival, and adaptation to hypoxic conditions (48, 49).

## Discussions

Gliomas are heterogeneous tumors originating from glial cells and remain the deadliest form of brain cancer. Despite significant research, the mechanisms by which glioma stem cell contribute to the diverse cellular composition of gliomas remain inadequately define (50). Although previous studies have revealed the involvement of multiple pathway alternations and genes, such as those involved in the Wnt pathway, the precise mechanisms driving glioma pathogenesis are still not fully understood (51). Advances in single-cell sequencing technologies have provided new insights into tumorigenesis, including glioma, from different aspects. For example, Ochocka et al. identified sex-specific gene expression in glioma-activated microglia that may influence the incidence and outcomes of glioma patients (52). Müller et al. profiled the human gliomas using single-cell RNA-seq and revealed macrophage ontogeny as a factor contributing to regional differences in macrophage activation within the tumor microenvironment (53). Venteiche et al. proposed a unifying model for IDH-mutant gliomas and a comprehensive framework for analyzing the differences across human tumor subclasses (54). The interaction pattern between GSC and immune cells during carcinogenesis demonstrated by Zhai et al. provides a theoretical basis for GSC-targeted immunotherapy (55). However, the ST analysis of primary glioma remained mainly unexplored.

Various subtypes of gliomas have been identified, including OPC-like, AC-like, MES-like and NPC-like. By integrating single-cell and spatial transcriptomics. we observed the distribution of these subtypes in glioma samples. We discovered that oxidative stress response genes play a vital role in the generation of OPC-like subtype. We observed increased expressions of oxidative stress response genes, heat-shock proteins, and DNA damage response genes throughout the evolutionary trajectory of OPC subclusters. These findings suggest that stress responses are activated and potentially enhance the gliomagenesis. Notably, the situation was different between HG and LG gliomas. In HG gliomas, the hypoxic state is associated with uncontrolled proliferation and subsequent malignancy in the tumor core. Especially for HG1, the EMT was observed in the core region. For HG2, the pathways altered were more about the proliferation and cell cycle regulation.

We especially focused on JUN (AP1) and related TFs that regulate apoptosis and cell survival in various types of cancer. Our results revealed that the expression of AP1 in scRNA-seq data was associated with the EMT and expression of abnormal proliferation markers, such as VEGFA and VIM. In ST data, AP1 is enriched in more malignant OPC subtype MES-like and correlated with hypoxia stress. Histological analysis, including H&E and IHC results, confirmed that these genes were expressed in the necrotic areas and glioma pseudo-palisade regions typically surround necrotic foci (53). Pseudo-palisades in glioblastoma are characterized by hypoxia, extracellular matrix protease expression, and an actively migrating cell population (54). These observations were corroborated by IVY, an anatomic transcriptional atlas of human glioblastoma. We used the RNA-Seq as well as the image data from a cellular resolution of ISH tissue sections and adjacent H&E stained sections annotated for anatomic structures and found that JUN (AP1) was highly expressed in necrotic and pseudopalisade regions. These findings implied that oxidative stress-induced expression of JUN-AP1 might impact gliomagenesis and malignant transformation. AP1, a well-known stress-responsive TF, responds to various stimuli, including cytokines, growth factors, and stress signals. It plays a key role in various cancers, including glioma, by regulating its downstream targets. For instance, inhibition of c-Jun N-terminal kinase enhances temozolomide-induced cytotoxicity in human glioma cells (55). FRK controls the migration and invasion of human glioma cells by regulating JNK/c-Jun signaling (56). Similar situations were found for c-FOS, a major component of the AP1 complex. Silencing c-Fos sensitized glioma cells to radiation by increasing radiation-induced DNA double-strand breaks (DSBs), disturbing the DNA damage repair process, promoting G2/M cell cycle arrest, and enhancing apoptosis (57). Our results implied that these TFs might activate in response to oxidative stress sounded by the necrotic areas and trigger the protective role in cancer cells.

Additionally, we observed a loss of mitochondrial genes in HG glioma samples, particularly in the malignant MES-like subtype. Mitochondria are critical for apoptosis activation in mammalian cells; thus, mitochondrial dysfunction is often in gliomas as a mechanism to evade apoptosis (58). Previous studies showed a pathway-based classification of glioblastoma associated with a mitochondrial subtype with therapeutic vulnerabilities (59). These results suggested that mitochondrial subtype in OPC-like might associated with its therapeutic results. For instance, oncogene AIF-2 regulates proliferation, migration, and invasion of human glioma cells via mitochondrial dysfunction (60). HIF-1α, a key regulator of metabolism, affects Tregs migration and immunosuppression by switching between glycolytic and oxidative phosphorylation pathways (61). Moreover, failed apoptosis has a specific transcriptional signature regulated by JNK (an upstream kinase of AP1) and is enriched in metastatic melanoma (62). Our results also showed that mitochondrial gene lost is associated with high hypoxia score in HG gliomas, accompanied by increased expression of EGFR and other proliferative markers. This suggests that stress response TFs might regulate mitochondrial genes.

Furthermore, we observed distinct heterogeneities between LG1 *vs* LG2 and HG1 *vs* HG2. LG2 contains more OPC-like malignant components, whereas LG1 has more AC-like malignant components. This suggests that different subtypes of gliomas may have distinct cellular hierarchies and molecular characteristics, potentially influenced by intrinsic variations in glioma stem cells or microenvironmental factors. Additionally, genes in HG2 are less active compared to HG1 regions, likely due to varying microenvironmental conditions and stress responses. HG1 is characterized by a hypoxic state and EMT, driving uncontrolled proliferation, while HG2 focuses more on proliferation and cell cycle regulation pathways. The rapid increase in JUNB 1 expression observed in HG2 regions could be linked to differential stress responses and microenvironmental factors, indicating that glioma cells in these regions might experience unique stress conditions that promote specific gene regulatory mechanisms to enhance survival and proliferation.

Taken together, for the first time based on the scRNA-seq and ST data, this study provides novel insights into oxidative stress-induced distinct pathways in HG and LG, JUN (AP1) might be a critical regulator in the tumorigenesis processes of OPC-like glioma under stress conditions.

Our study, while comprehensive, has several limitations. Firstly, the combination of single-cell RNA sequencing (scRNA-seq) and spatial transcriptomics (ST) provided valuable insights, but the spatial resolution might not fully capture the complex cellular interactions and heterogeneity within gliomas. Additionally, our findings are based on a limited number of high-grade (HG) and low-grade (LG) glioma samples, which restricts the generalizability of the results across different glioma subtypes and patient demographics. The cross-sectional nature of our data further limits the understanding of temporal changes during gliomagenesis and the progression from low-grade to high-grade gliomas, necessitating longitudinal studies to capture these dynamic processes. Furthermore, our study primarily relies on transcriptomic data to infer gene function and pathway activation, underscoring the need for functional validation through *in vitro* and *in vivo* experiments to confirm the roles of identified genes and pathways in glioma progression. Lastly, while we identified potential therapeutic targets such as JUN (AP1), the translational aspect of these findings requires further investigation, including drug efficacy studies and clinical trials.

To build on our findings, several future directions can be pursued. Enhancing spatial resolution using advanced technologies can provide more detailed insights into the cellular architecture and interactions within the glioma microenvironment. Integrative multi-omics approaches that combine transcriptomics with proteomics, metabolomics, and epigenomics can offer a more holistic view of the molecular mechanisms driving gliomagenesis and progression. Conducting longitudinal studies to monitor the temporal evolution of gliomas and the impact of therapeutic interventions will be crucial for understanding disease dynamics and treatment responses. Functional studies, including gene knockdown or overexpression experiments and the use of animal models, are necessary to validate the roles of key genes and pathways identified in our study. Investigating the clinical relevance of identified biomarkers and therapeutic targets, including their predictive value for patient prognosis and response to treatment, will be a critical step towards translating these findings into clinical practice. Exploring the interactions between glioma cells and their microenvironment, including immune cells, stromal cells, and extracellular matrix components, will provide insights into the mechanisms of tumor progression and resistance to therapy. Lastly, developing targeted therapies that modulate stress response pathways and metabolic shifts identified in our study could offer new treatment strategies for glioma patients.

## Data Availability

The data, presented in the study are deposited in the GEO repository, accession number GSE270355.

## Supplementary material

Supplementary Table 1 Baseline information of two patients in this study.
