# Supplementary material for: Single-cell and spatial transcriptome assays reveal heterogeneity in gliomas through stress responses and pathway alterations
Source: Front Immunol. 2024 Aug 27;15:1452172. doi: 10.3389/fimmu.2024.1452172 (PMC11385306; doi:10.3389/fimmu.2024.1452172)
Supplement: Supplementary file 1 [file DataSheet1.docx]

**Supplementary Figures and Figure Legends**


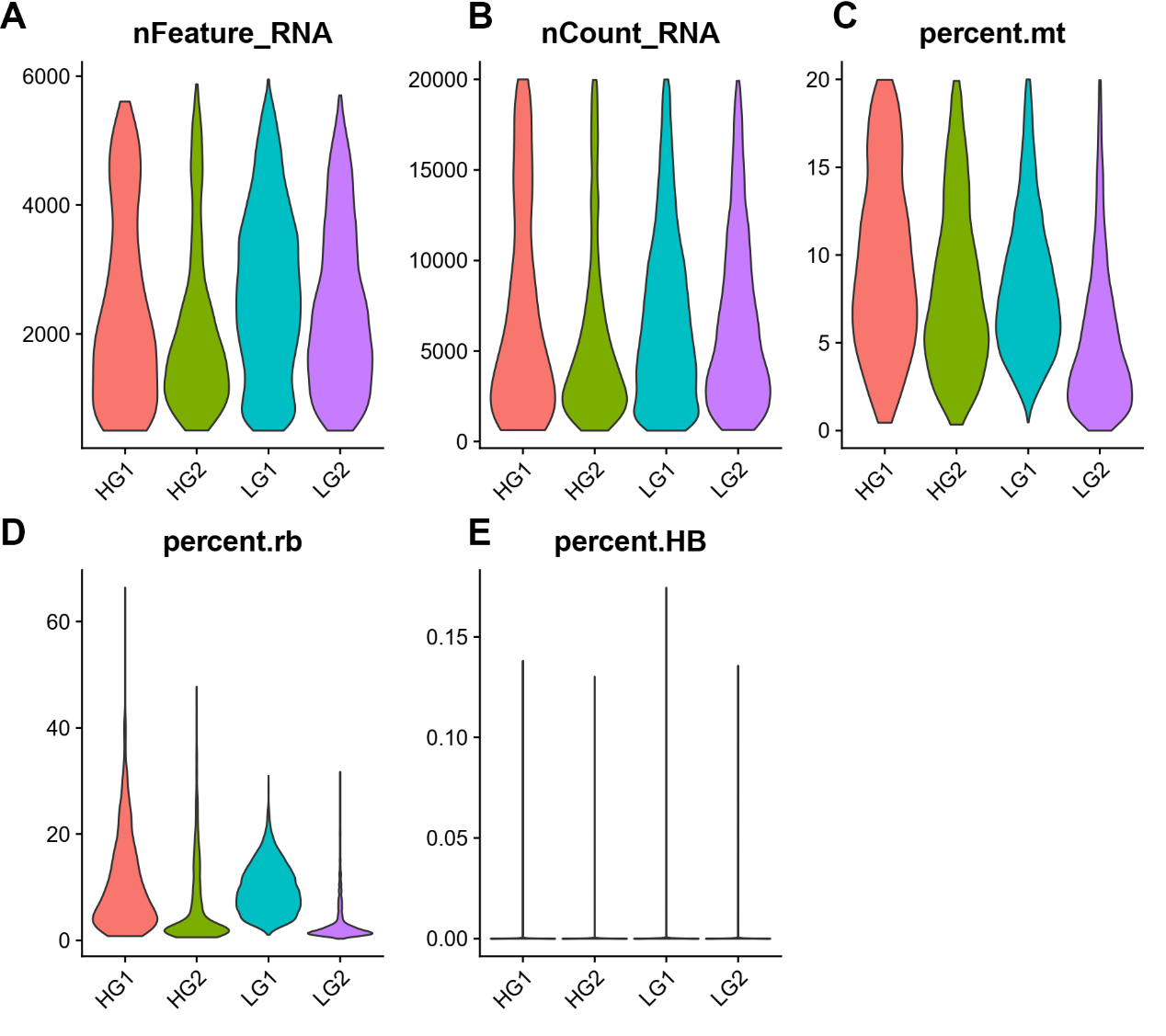


**Figure S1 Violin plots illustrating the expression distribution from scRNA-seq data.** The number of feature RNA (A), Count RNA (B), percentage of mitochondrial genes (C), percentage of ribosome protein genes (D) and hemoglobin genes (E) from each sample were shown.

**
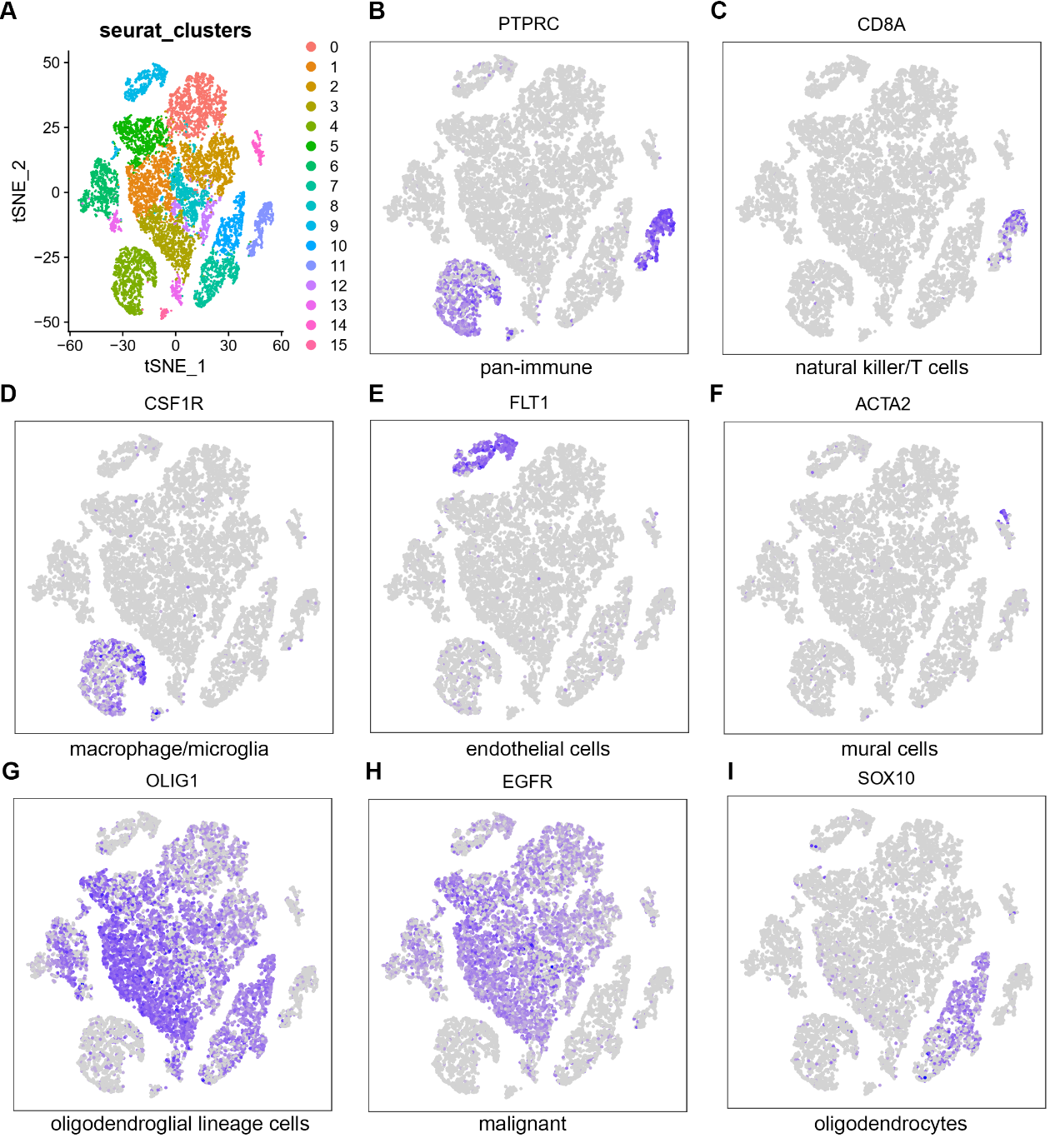
**

**Figure S2 Expression of presentative marker genes from each cluster of scRNA-seq data.** (A) t-distributed stochastic neighbor embedding (tSNE) projection of cells showing 16 major scRNA-seq clusters. (B-J) Feature tSNE projection depicting cluster-specific expression of *PTPRC* (Protein tyrosine phosphatase receptor type C), *CD8A* (Cluster of Differentiation 8a), *CSF1R* (Colony Stimulating Factor 1 Receptor), *FLT1* (Fms Related Receptor Tyrosine Kinase 1), *ACTA2* (Actin alpha 2, smooth muscle), *OLIG1* (Oligodendrocyte transcription factor 1), *EGFR* (Epidermal growth factor receptor) and *SOX10* (SRY-Box Transcription Factor 10). The predicted type of each cell cluster was indicated below.


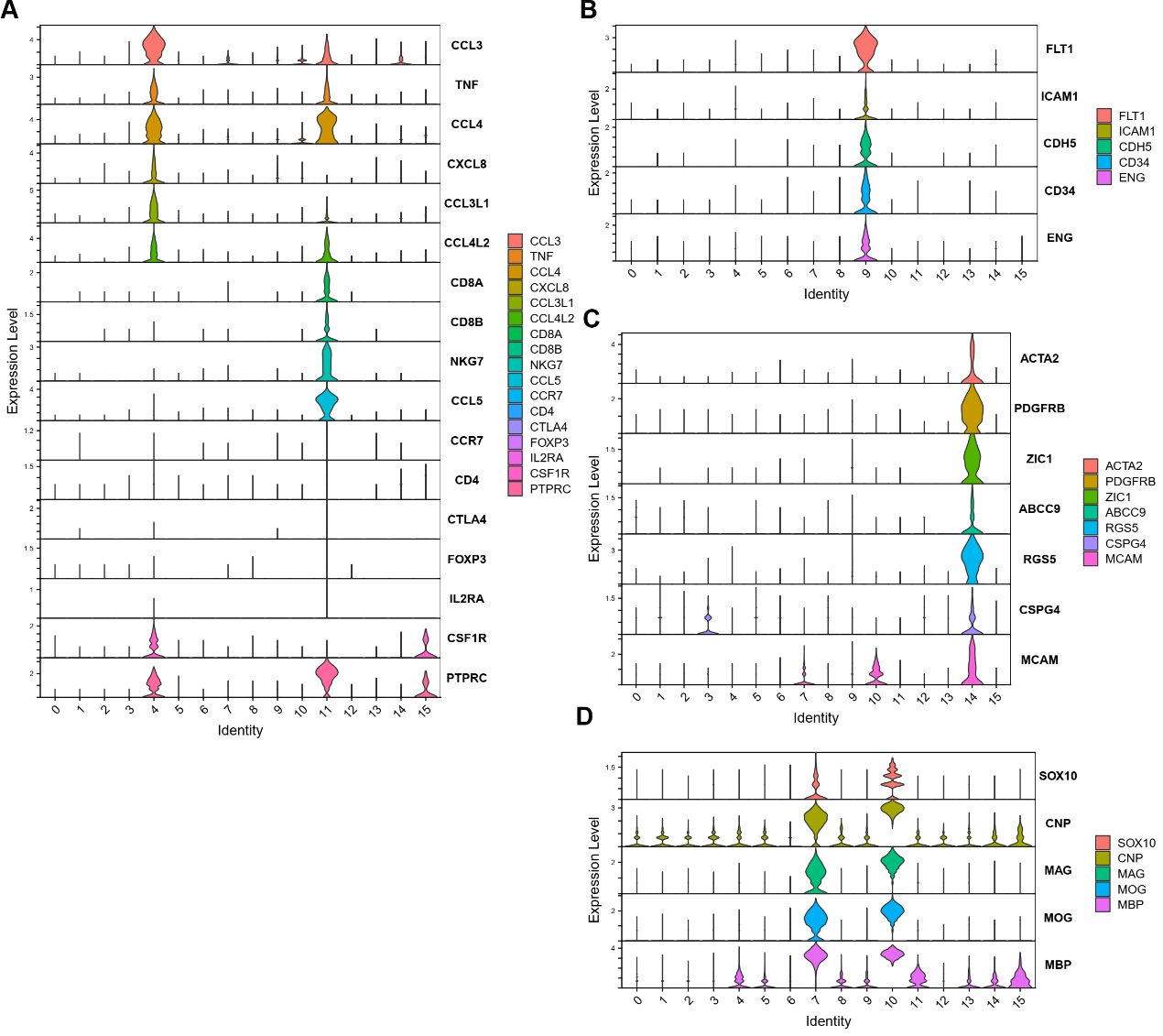


**Figure S3 Marker expressions for each cluster of scRNA-seq data**. Stacked violin plot for markers from normal cell clusters including immune cell group (A), endothelial cells (B), mural cells (C) and oligodendrocytes (D).


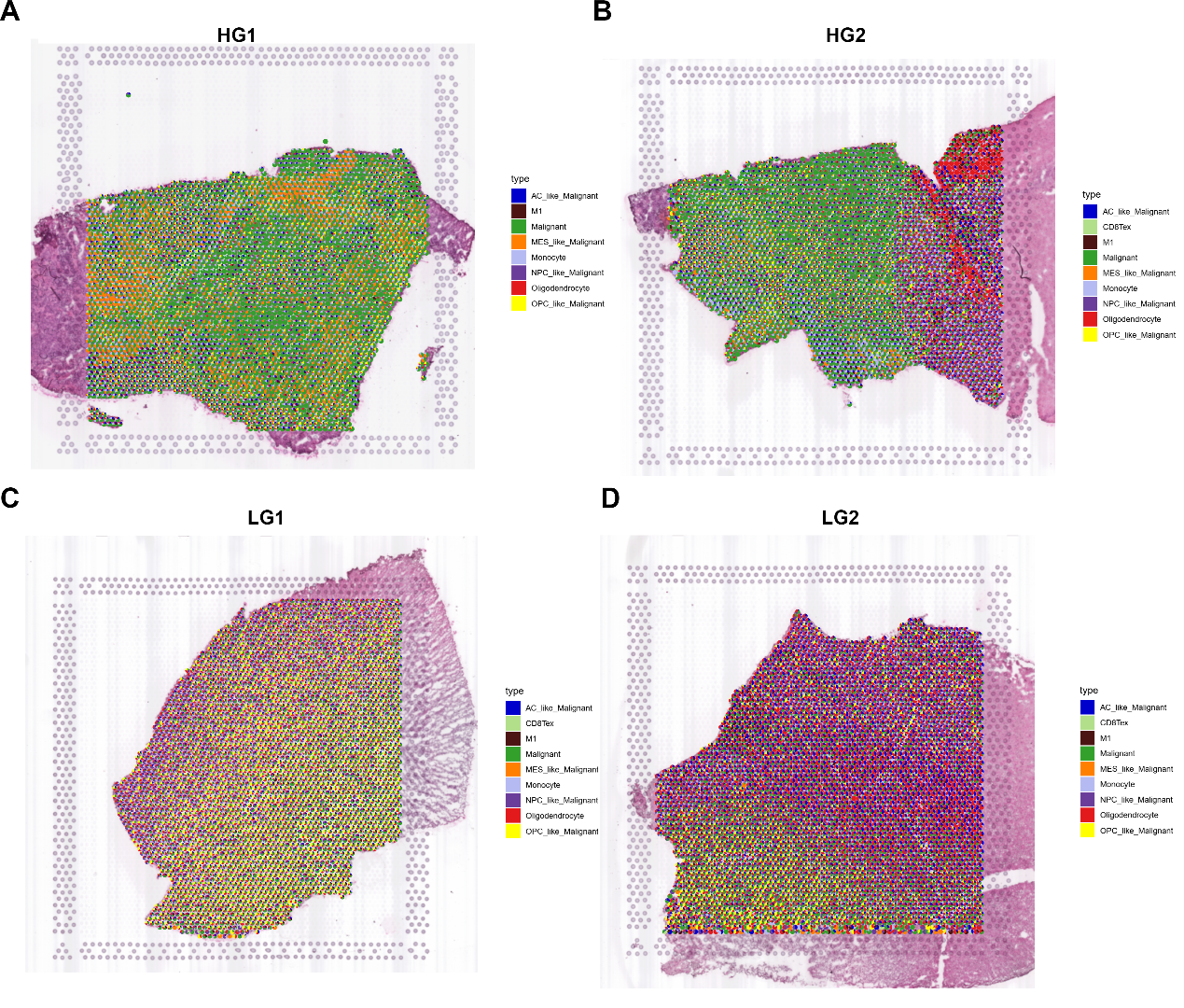


**Figure S4 ST annotations by different types. (**A-D**)** The different types of spots were annotated according to the expression patterns including AC-like malignant, M1, Malignant, MES-like malignant, Monocyte, NPC-like malignant, OPC-like malignant.


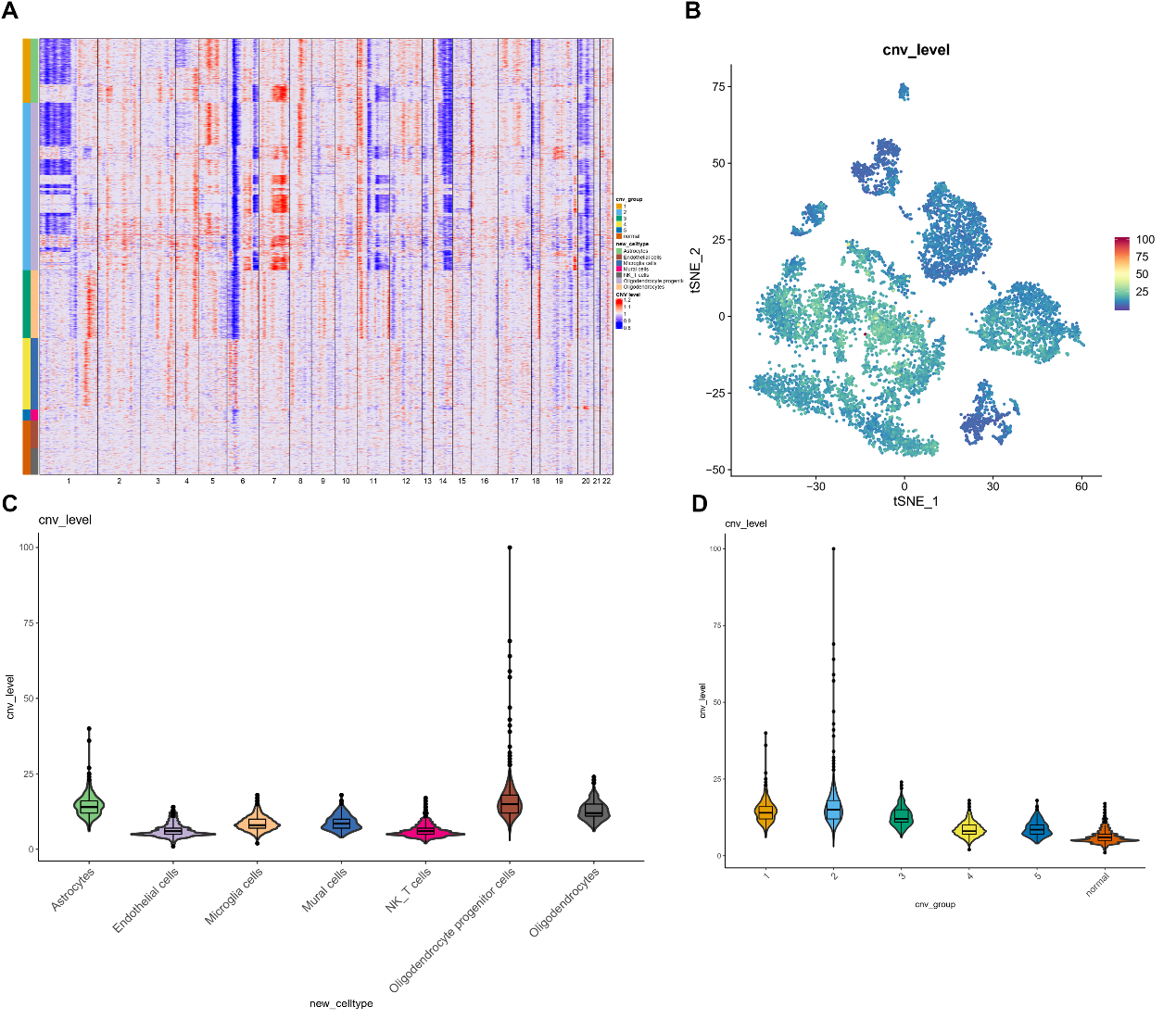


**Figure S5 The copy number variation of different cell types from scRNA-seq.** The infercnv package was used to predict the copy number variation among different cell types. (A) Heatmap of the estimated copy number (ECN) of all chromosomes (columns) in scRNA-seq data. (B) Projection (UMAP) plot showing estimated CNV numbers. (C) Violin plot showing that average CNV levels from each different type of cells. (D) Violin plot showing the average CNV levels from 5 different CNV type subclusters.


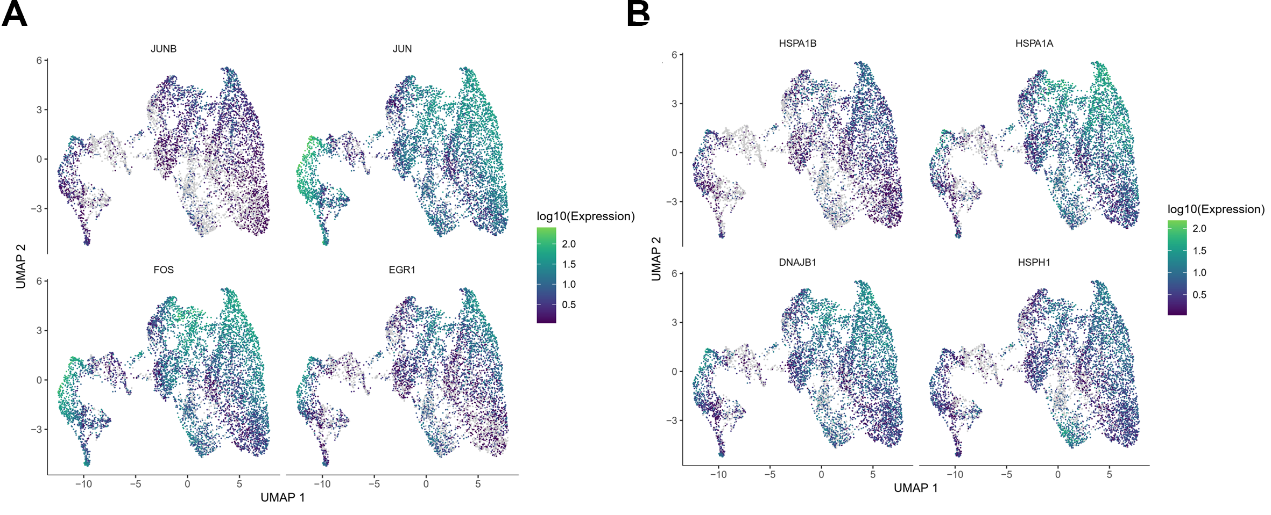


**Figure S6 Stress response proteins are activated in the gliomagenesis trajectory of OPC subcluster** (A) UMAP projection of OPC subclusters showing the expressions of transcription factors that involved in the oxidative stress response such as *JUN, JUNB, FOS* and *EGR1*. (B) UMAP projection of OPC subclusters showing the expressions of heat shock proteins and DNA-damage response proteins including HSPA1B, HSPA1A, DNAJB1 and HSPH1.


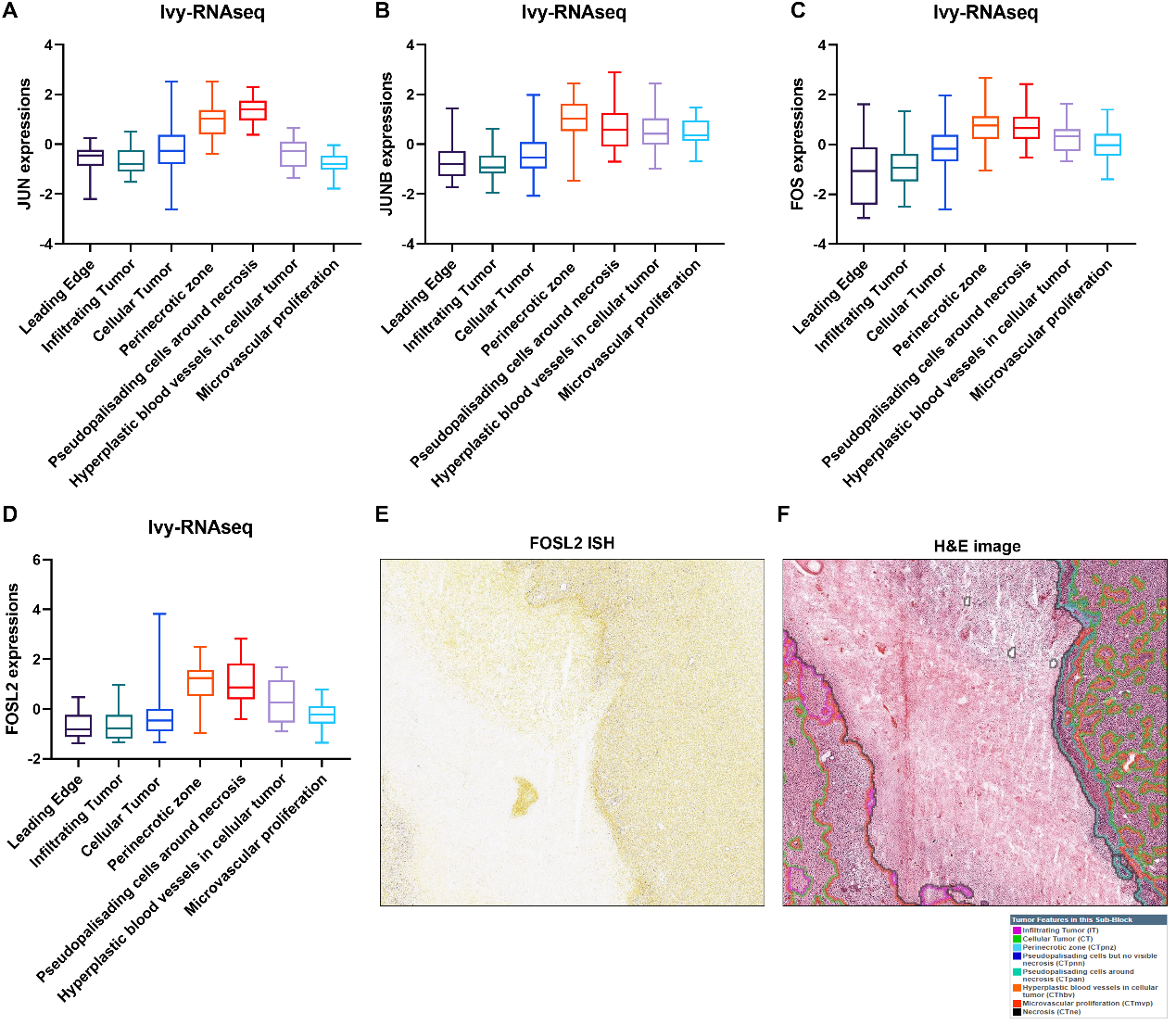


**Figure S7 Highly expression of JUN around the necrotic and pseudopalisading region in glioma.** (A-D) Specific mRNA expressions of *JUN*, *JUNB*, *FOS* and *FOS2L* were shown with according to the different tumor feature annotations including leading edge, infiltration tumor, cellular tumor, peri-necrotic zone, pseudopalisading cells around necrosis, hyperplastic blood vessels in cell tumor and microvascular proliferation. (E) Labeled using in situ hybridization (ISH) probe against FOS2L and (F) reference H&E section from tumor W1-1-2-Z.1.01 with feature annotation in the Ivy Glioblastoma Atlas Project. Scale bar, 1000 μm. FOS like 2, AP-1 transcription factor subunit.


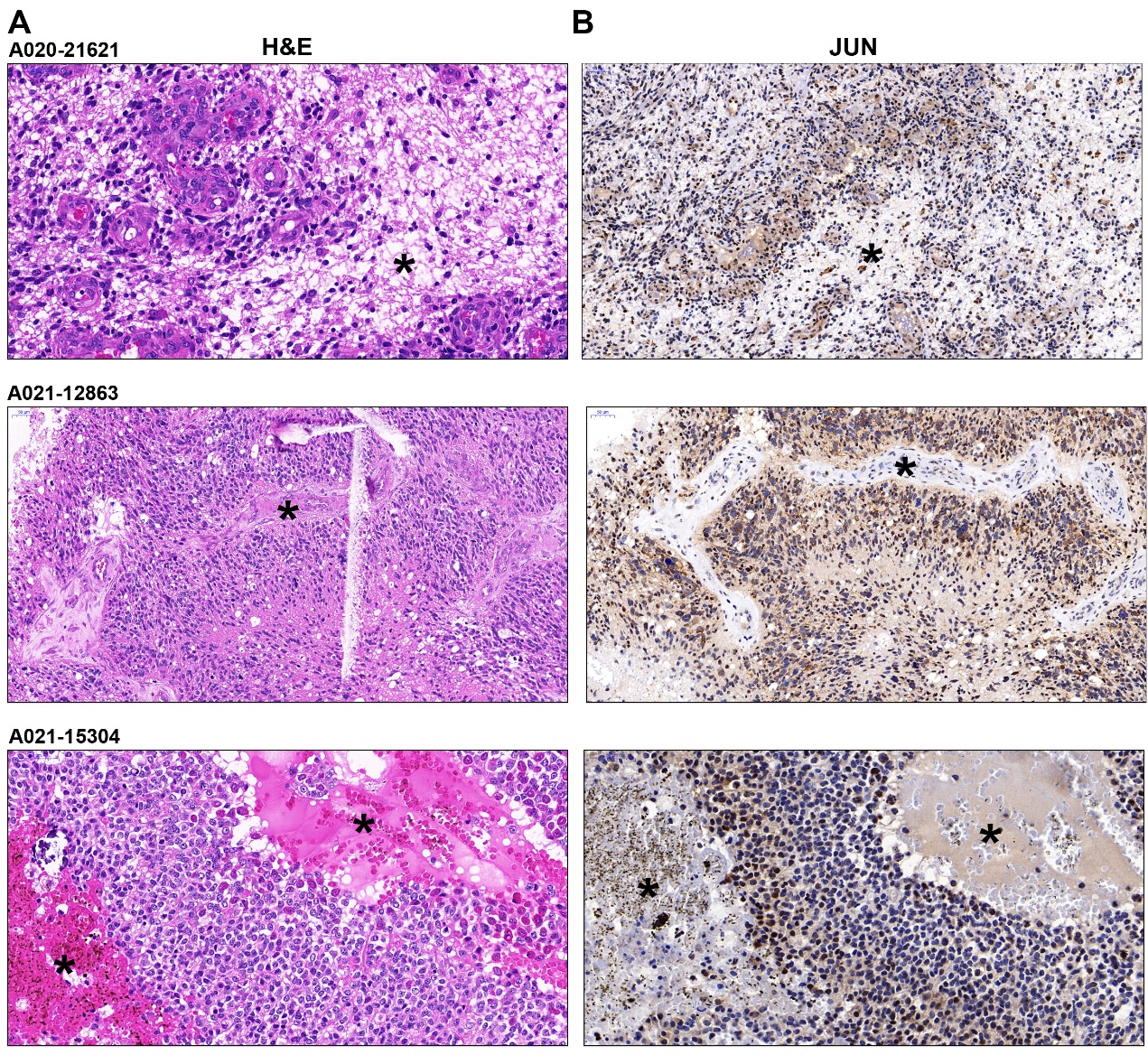


**Figure S8 Highly expression of JUN around the necrotic and pseudopalisading region in primary glioma samples.** (A) H&E staining for three independent glioblastoma samples (A020-21621, A021-12863, A021-15304). (B) The IHC staining for JUN showing the specific elevation of its protein level around the necrotic and pseudopalisading region in glioma samples. All the pictures were taken under 20x magnifications. * indicated necrotic and pseudopalisading region.


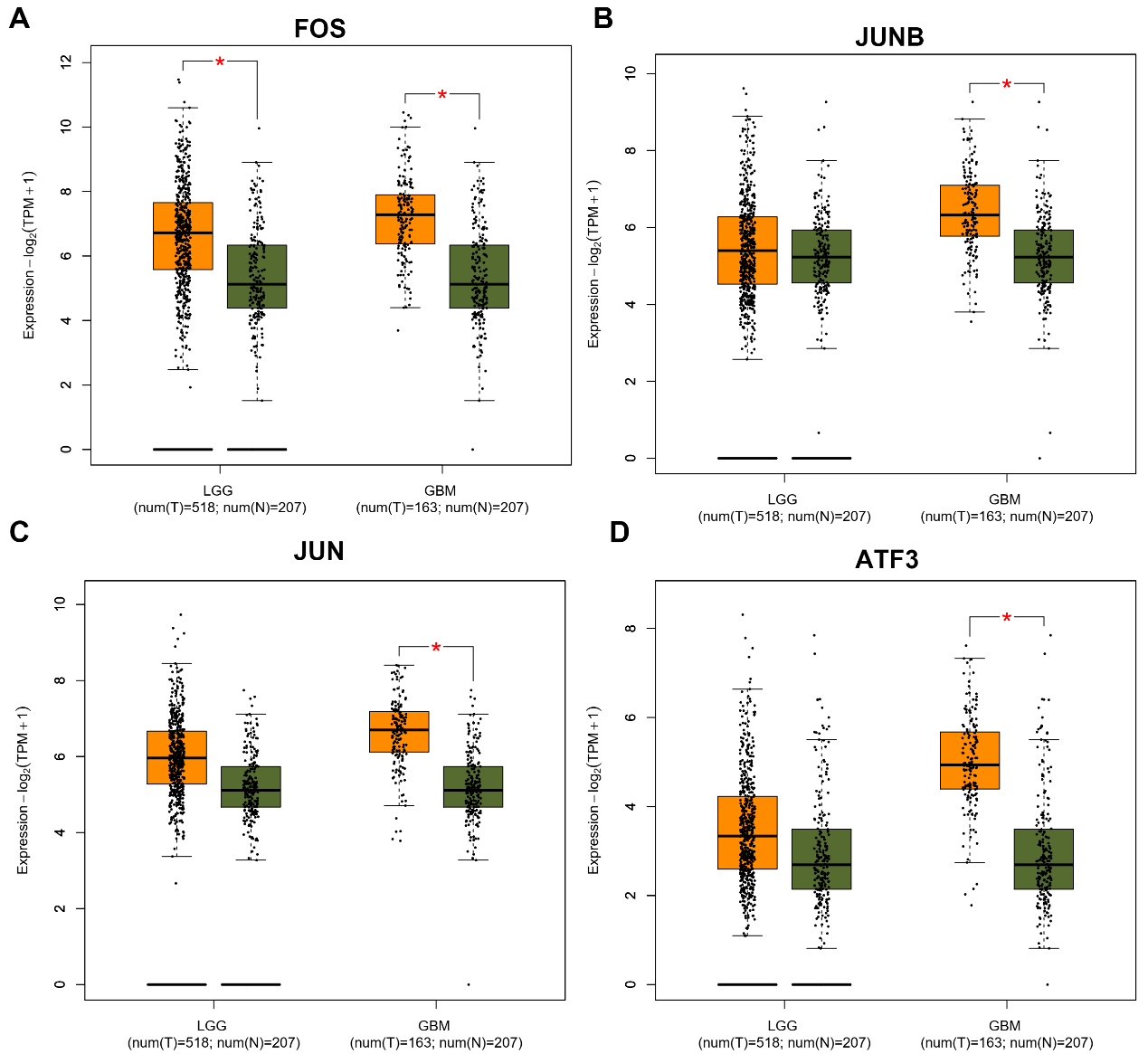


**Figure S9 Elevated expression of JNK-AP1 family genes in glioma samples.** (A-D) Gene Expression Profiling Interactive Analysis (GEPIA) was performed to validate the differential expression of four stress response genes (*FOS*, *JUNB*, *JUN* and *ATF3*) in glioma samples compared with normal samples in TCGA-GBM/LGG and Genotype-Tissue Expression (GTEx) dataset. The expression level from GEPIA was presented as log2(TPM+1). Orange box was the cancer tissue group, grass green was the normal tissue group, and asterisk represented p < 0.01. The dots represented expression in each sample.


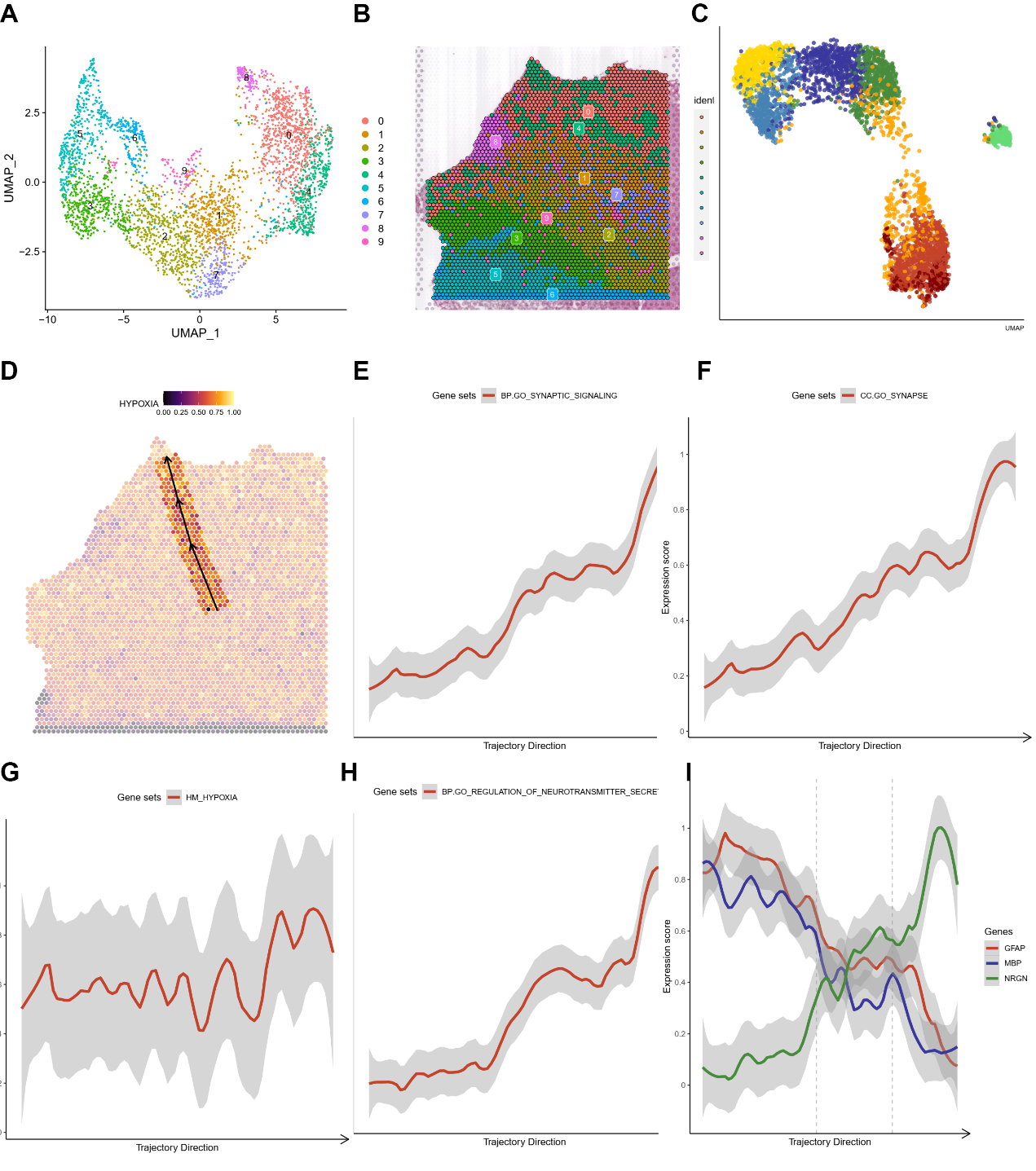


**Figure S10** **The hypoxia pathway trajectory with Monocle3 in LG1** (A) UMAP plot showing independent 7 major ST clusters. (B) The distribution of each spatial clusters in the ST profile from HG2 tissues. (C) The UMAP plot for spatital clusters using SPATA package. (D) The hypoxia trajectory for HG2. (E-H) The altered pathways in the trajectory. (I) The expressions of marker genes (JUN and FOSL2) in the trajectory.


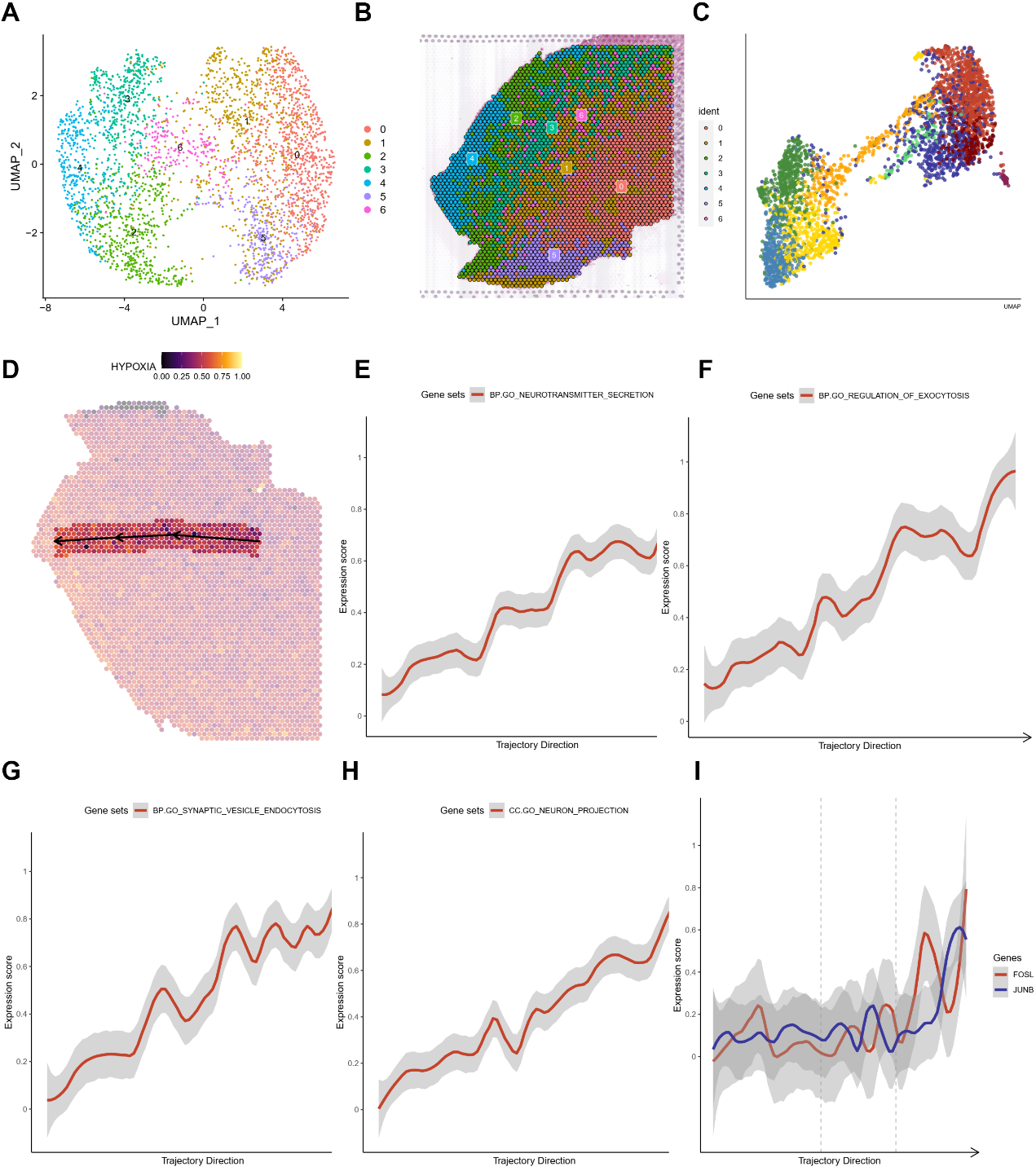


**Figure S11** **The hypoxia pathway trajectory with Monocle3 in LG2** (A) UMAP plot showing independent 7 major ST clusters. (B) The distribution of each spatial clusters in the ST profile from HG2 tissues. (C) The UMAP plot for spatital clusters using SPATA package. (D) The hypoxia trajectory for HG2. (E-H) The altered pathways in the trajectory. (I) The expressions of marker genes (JUN and FOSL2) in the trajectory.


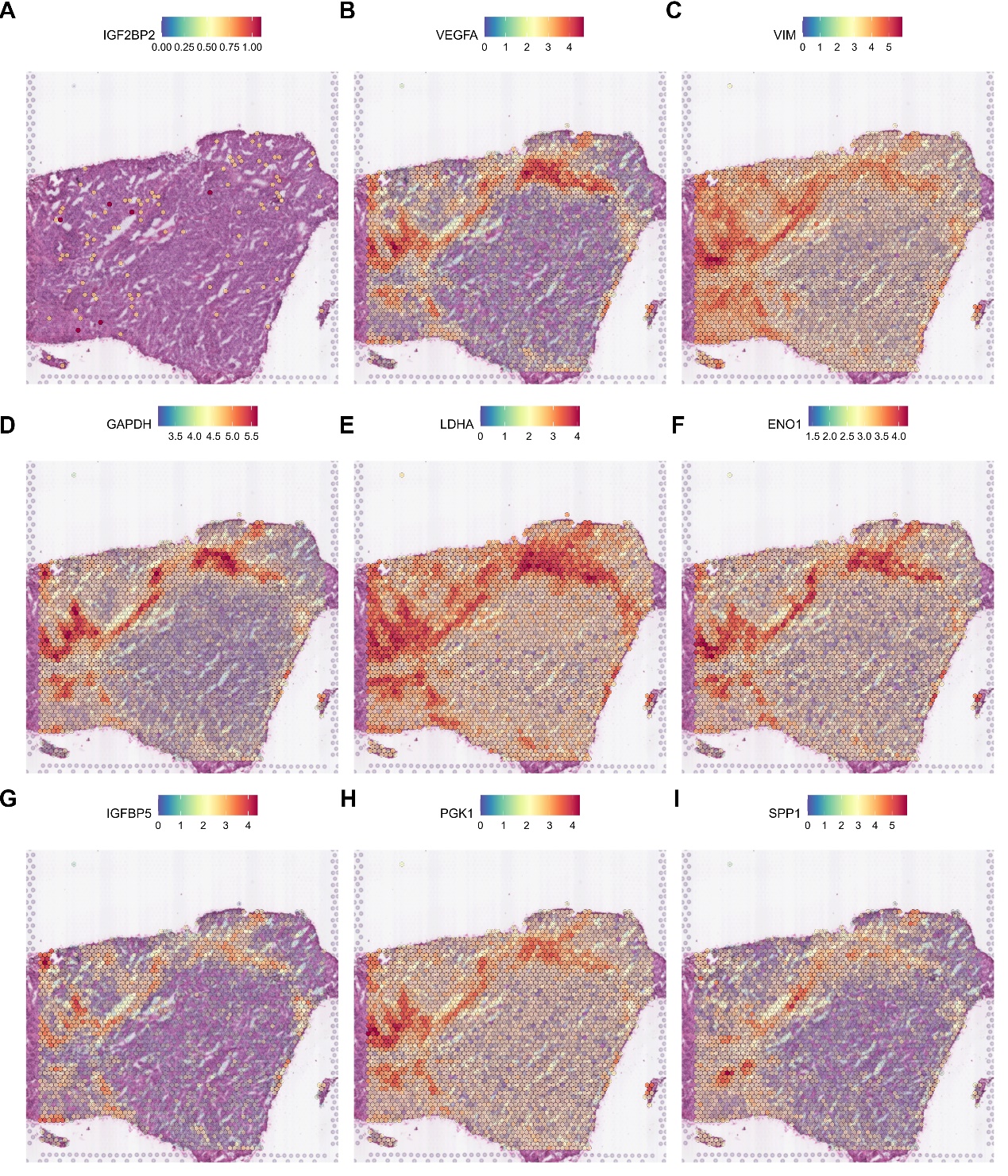


**Figure S12 Expression patterns of presentative marker genes for tumor proliferation and metabolism in HG1 spatial transcriptomics data.** (a-f) The expressions of top marker genes including (*IGFBP2, VEGFA, VIM, GAPDH, LDHA, ENO1, IGFBP5, PGK1 and SPP1*) was shown in the ST profile from HG1 tissues.


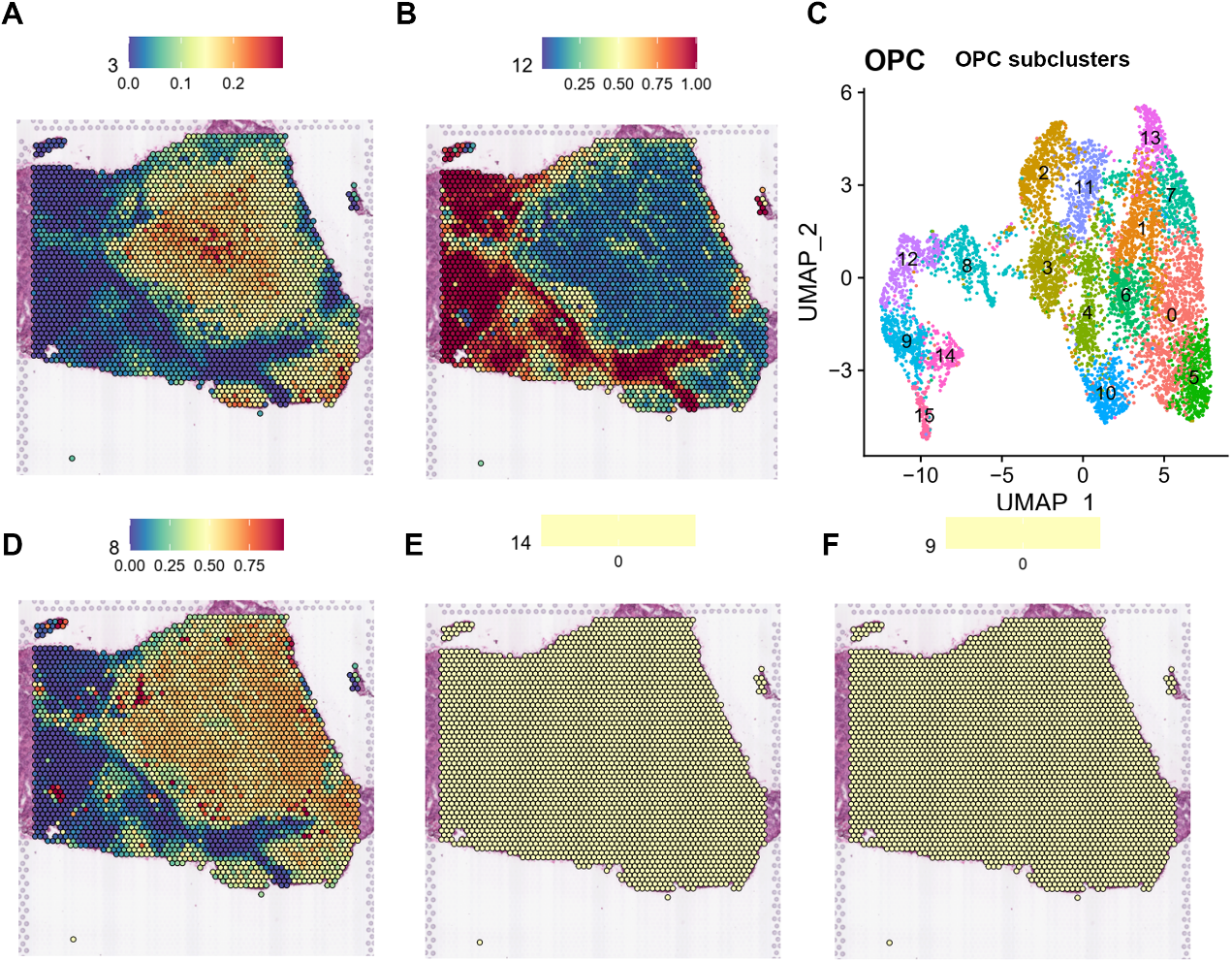


**Figure S13 The** **spatial relationship among different OPC subclusters in HG1** (A, B, D-F) The subclusters of OPC The projection of major OPC subcluster into the ST profiles from HG1**.** (C) The UMAP plot with different OPC subclusters was shown.


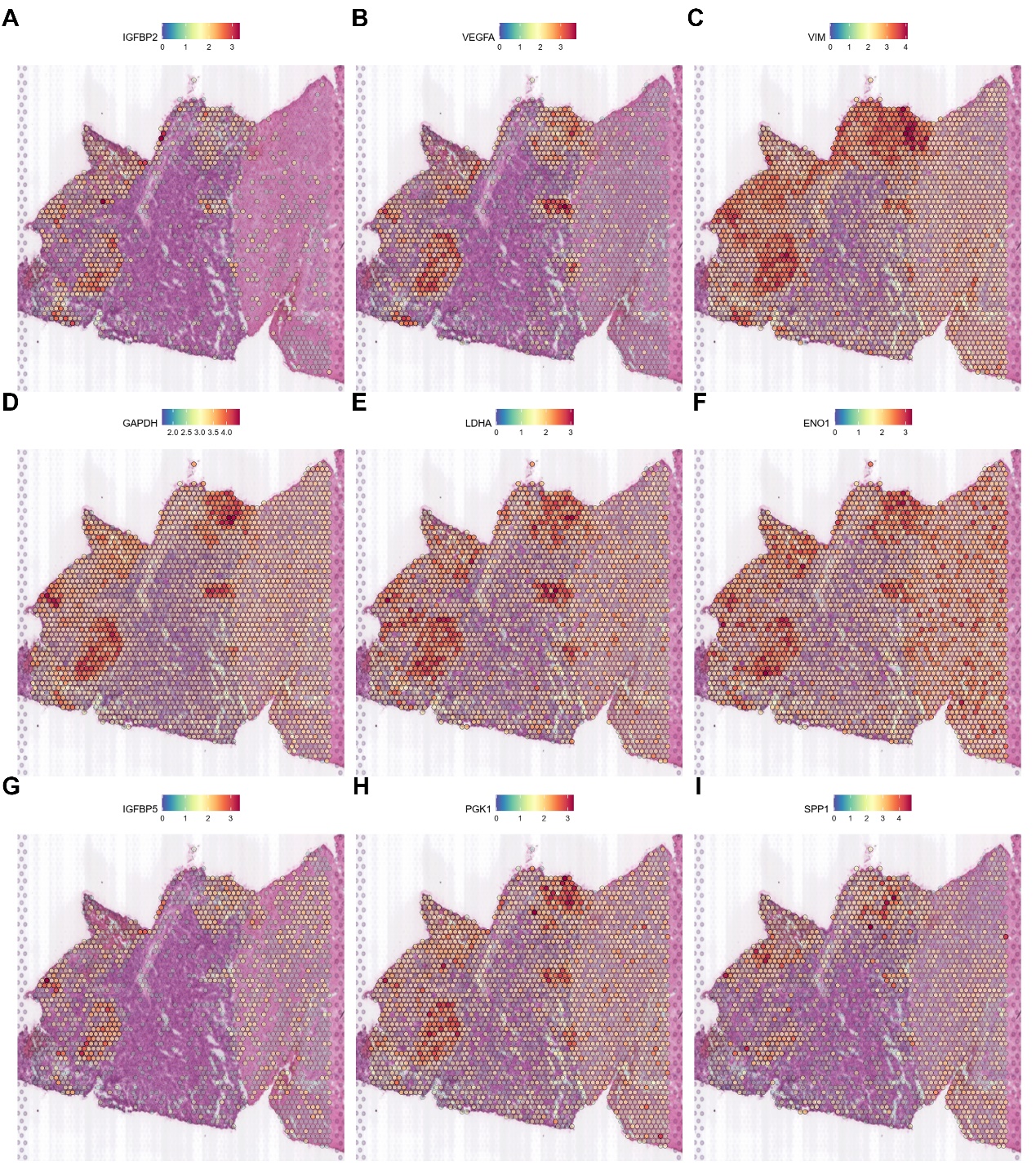


**Figure S14 Expression patterns of presentative marker genes for tumor proliferation and metabolism in HG2 spatial transcriptomics data.** (A-I) The expressions of top marker genes including (*IGFBP2, VEGFA, VIM, GAPDH, LDHA, ENO1, IGFBP5, PGK1 and SPP1*) was shown in the ST profile from HG2 tissues.


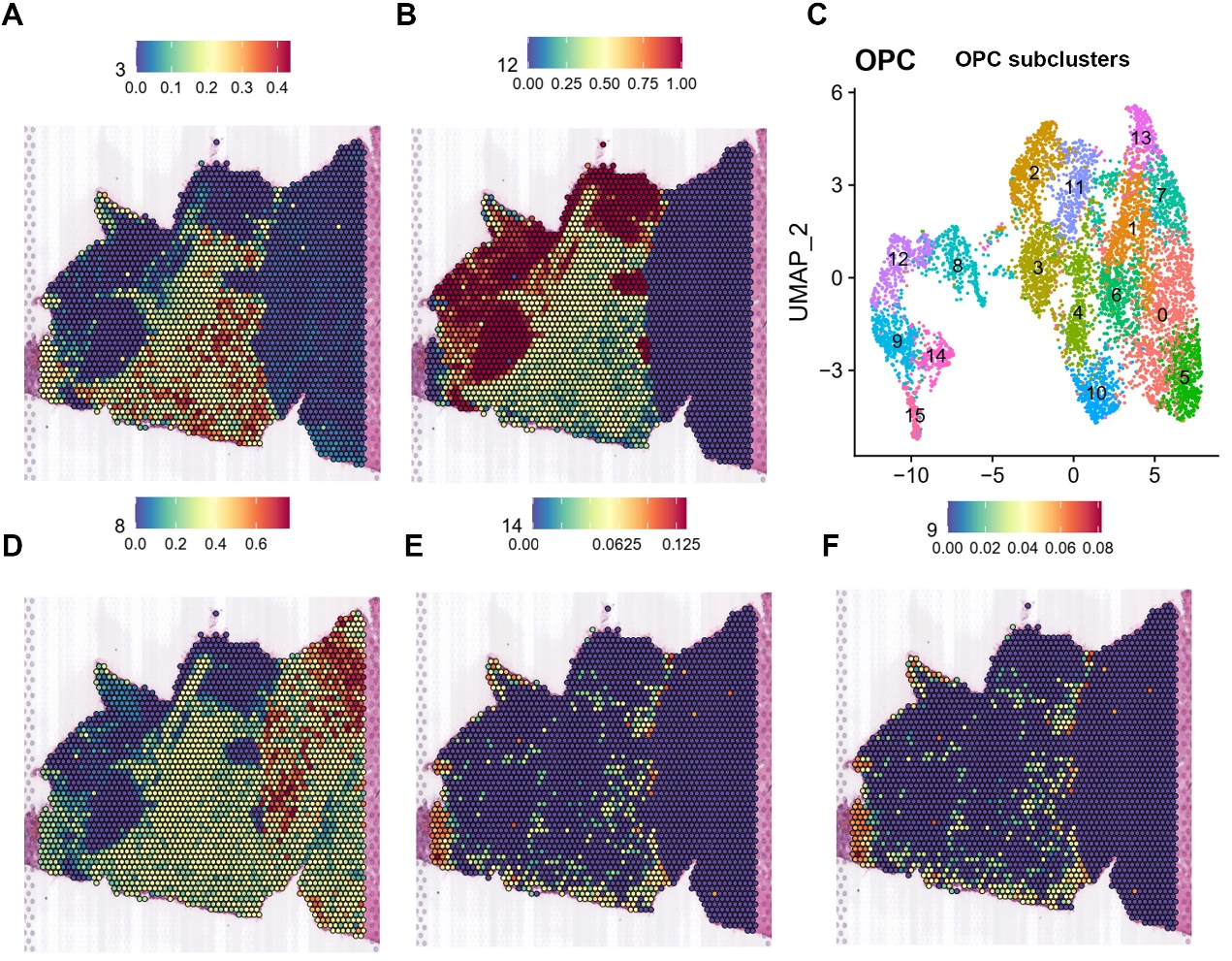


**Figure S15 The** **spatial relationship among different OPC subclusters in HG2** (A, B, D-F) The subclusters of OPC The projection of major OPC subcluster into the ST profiles from HG2. (C) The UMAP plot with different OPC subclusters was shown.


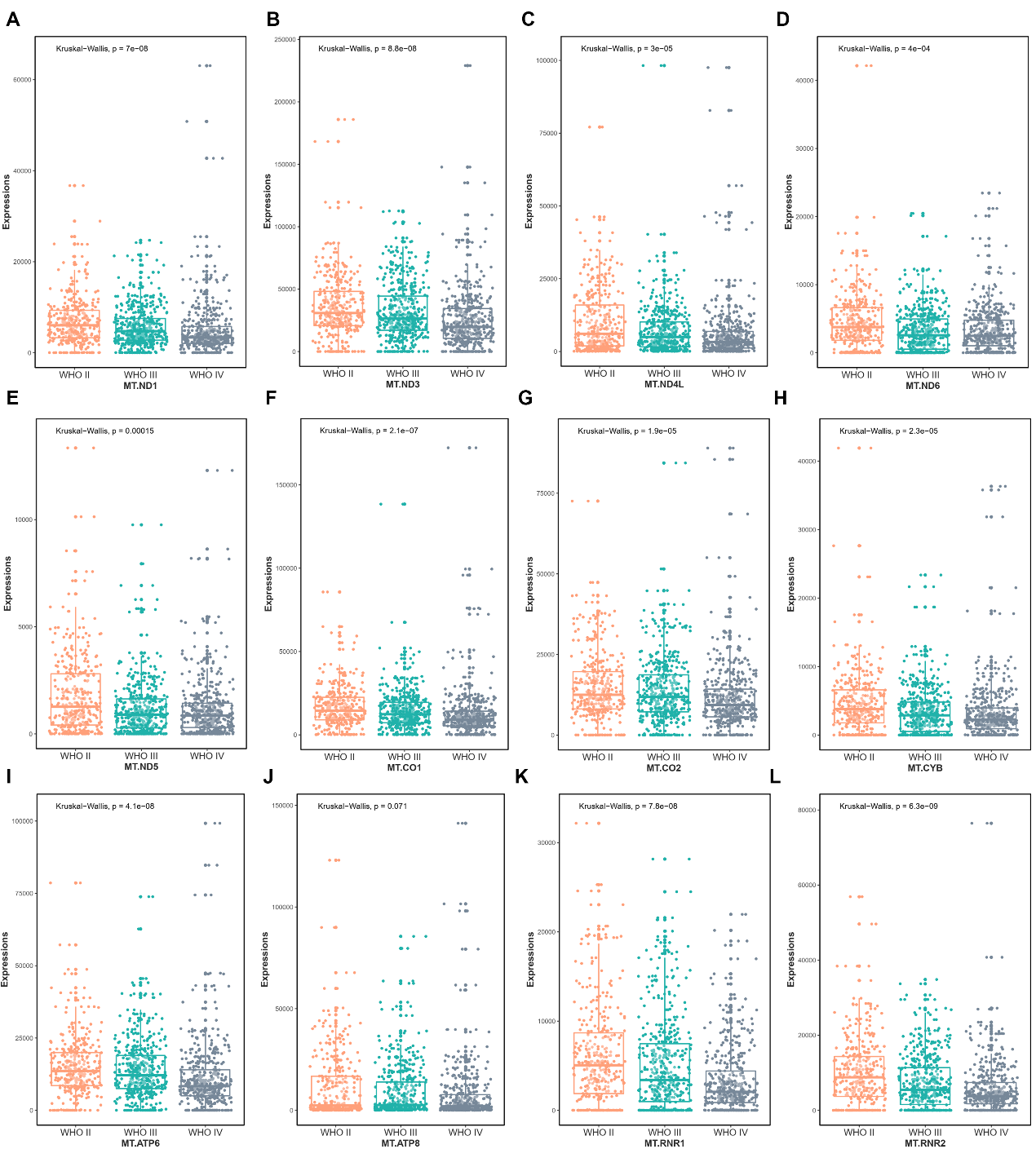


**Figure S16 Down-regulation of mitochondrial genes with WHO grade in CGGA glioma dataset.** (A-L) Correlation of mitochondrial genes (*MT-ND1*, *MT-ND3, MT-ND4L*, *MT-ND6*, *MT-ND5*, *MT-CO1*, *MT-CO2*, *MT-CYB*, *MT-ATP6*, *MT-ATP8*, *MT-RNR1* and *MT-RNR2*) transcription levels with glioma WHO grade.


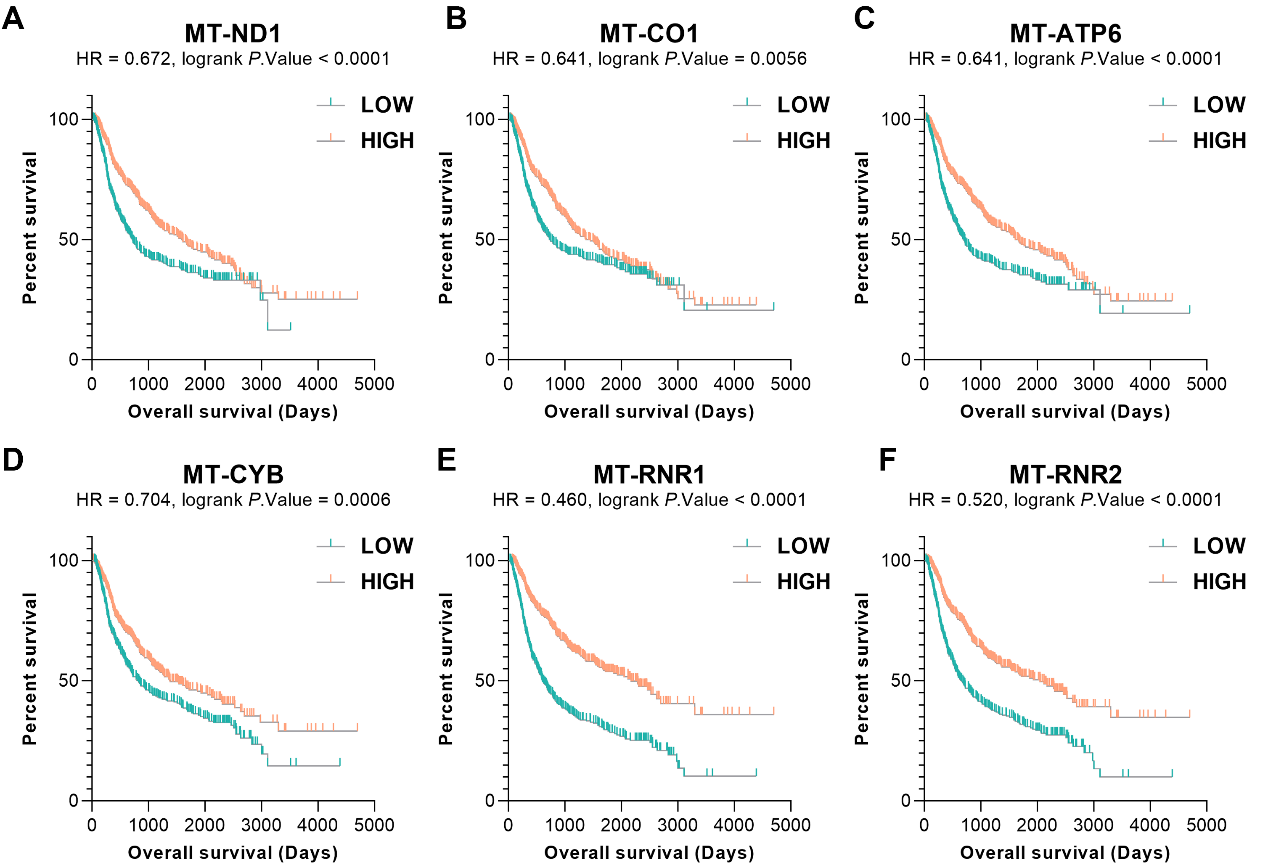


**Figure S17 Mitochondrial genes as a protective factor associated with overall survival in CGGA cohort.** (A-F) Kaplan-Meier analyses comparing overall survival between groups expressing higher and lower levels of mitochondrial genes (*MT-ND1*, *MT-CO1*, *MT-ATP6*, *MT-CYB*, *MT-RNR1*, *MT-RNR2*). The hazard ratio (HR) was calculated based on proportional hazards model. Data are derived from CGGA dataset mRNAseq_693 (n = 693).


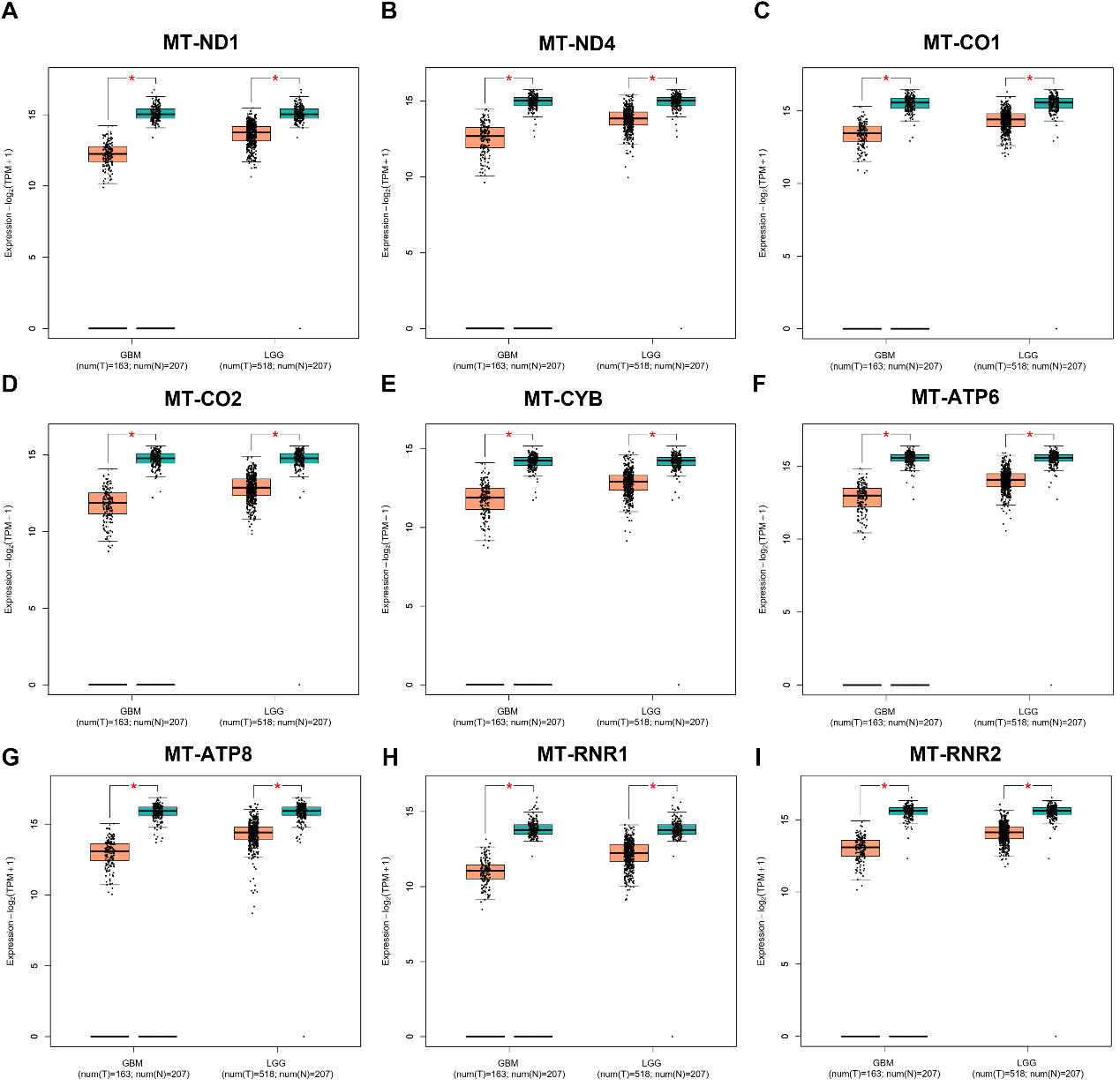


**Figure S18 Suppression expression of Mitochondrial genes in glioma samples.** (A-I) Gene Expression Profiling Interactive Analysis (GEPIA) was performed to validate the differential expression of mitochondrial genes (*MT-ND1*, *MT-ND4*, *MT-CO1*, *MT-CO2*, *MT-CYB*, *MT-ATP6*, *MT-ATP8*, *MT-RNR1* and *MT-RNR2*) in glioma samples compared with normal samples in TCGA GBM/LGG and GTEx dataset. The expression level from GEPIA was presented as log2(TPM+1). Orange box was the cancer tissue group, grass green was the normal tissue group, and asterisk represented p < 0.01. The dots represented expression in each sample.


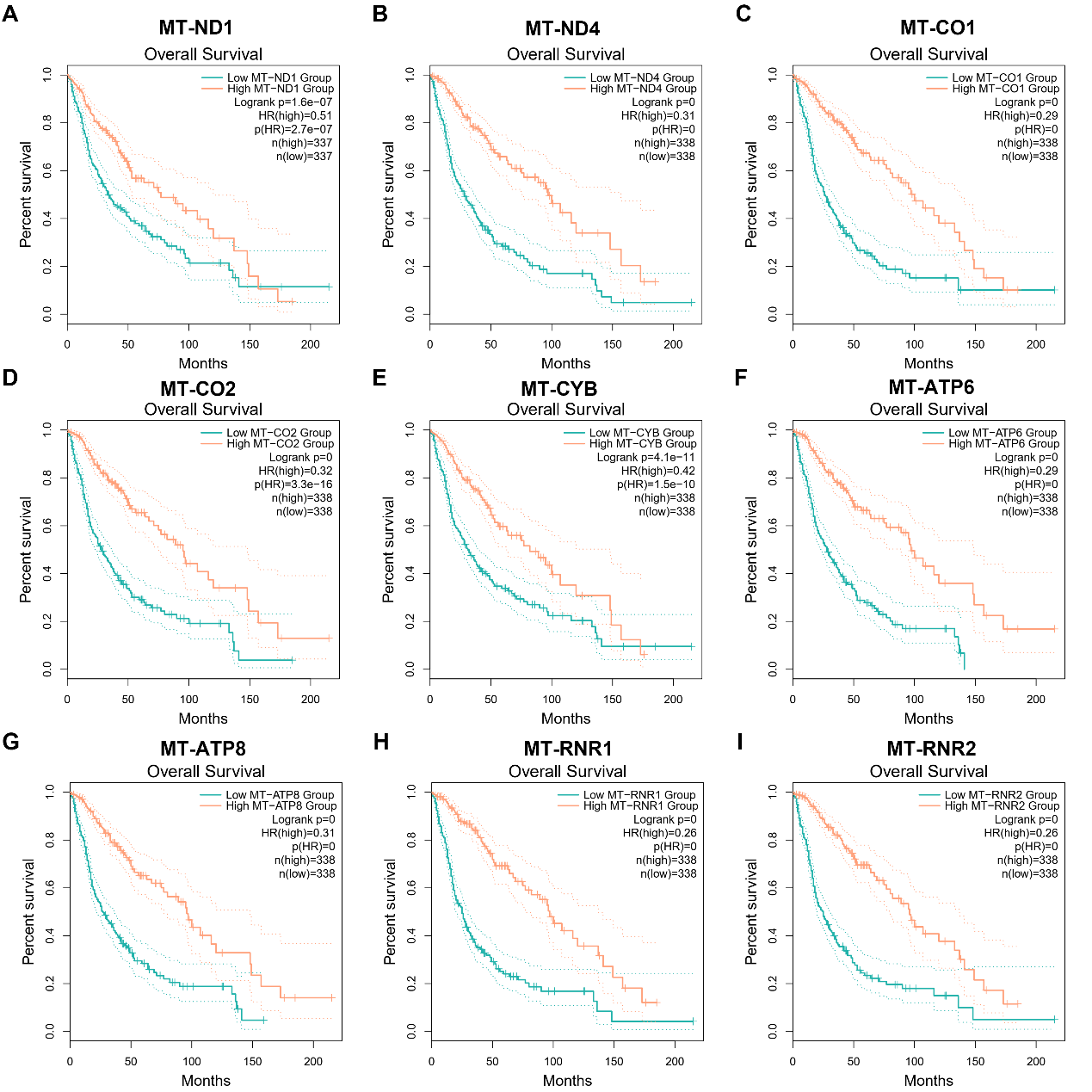


**Figure S19 Mitochondrial genes as a protective factor associated with overall survival in TCGA cohort.** (A-I) Kaplan-Meier analyses comparing overall survival between groups expressing higher and lower levels of mitochondrial genes (*MT-ND1*, *MT-ND4*, *MT-CO1*, *MT-CO2*, *MT-CYB*, *MT-ATP6*, *MT-ATP8*, *MT-RNR1* and *MT-RNR2*). The hazard ratio (HR) was calculated based on Cox's proportional hazards model. Data are from patients with TCGA GBM/LGG from GEPIA2.


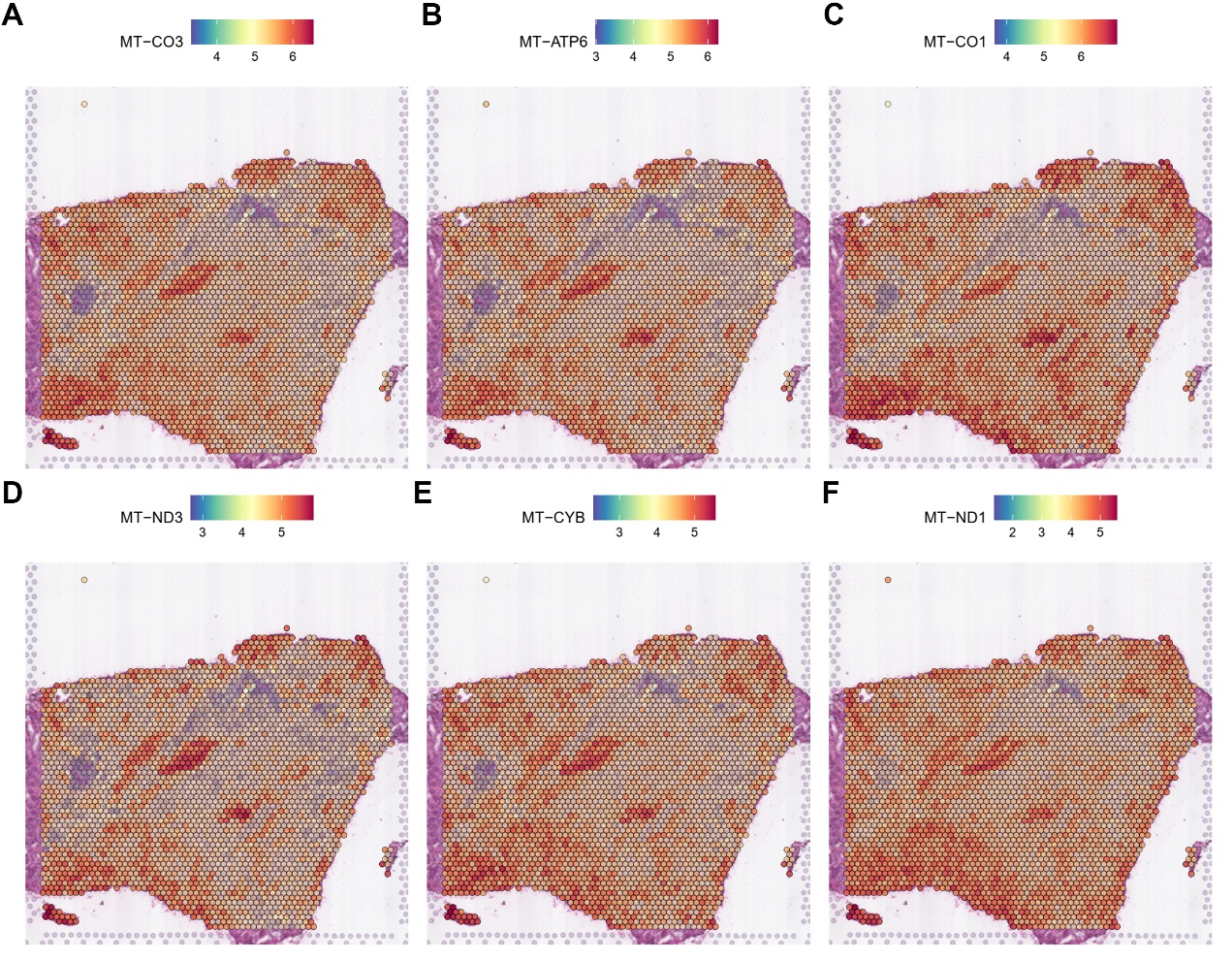


**Figure S20 Expression patterns of mitochondrial genes in HG1 spatial transcriptomics data**

(A-F) The expressions of mitochondrial genes including *MT-CO3, MT-ATP6, MT-CO1, MT-ND1, MT-CYB, MT-ND1* was shown in the ST profile from HG1 tissues.


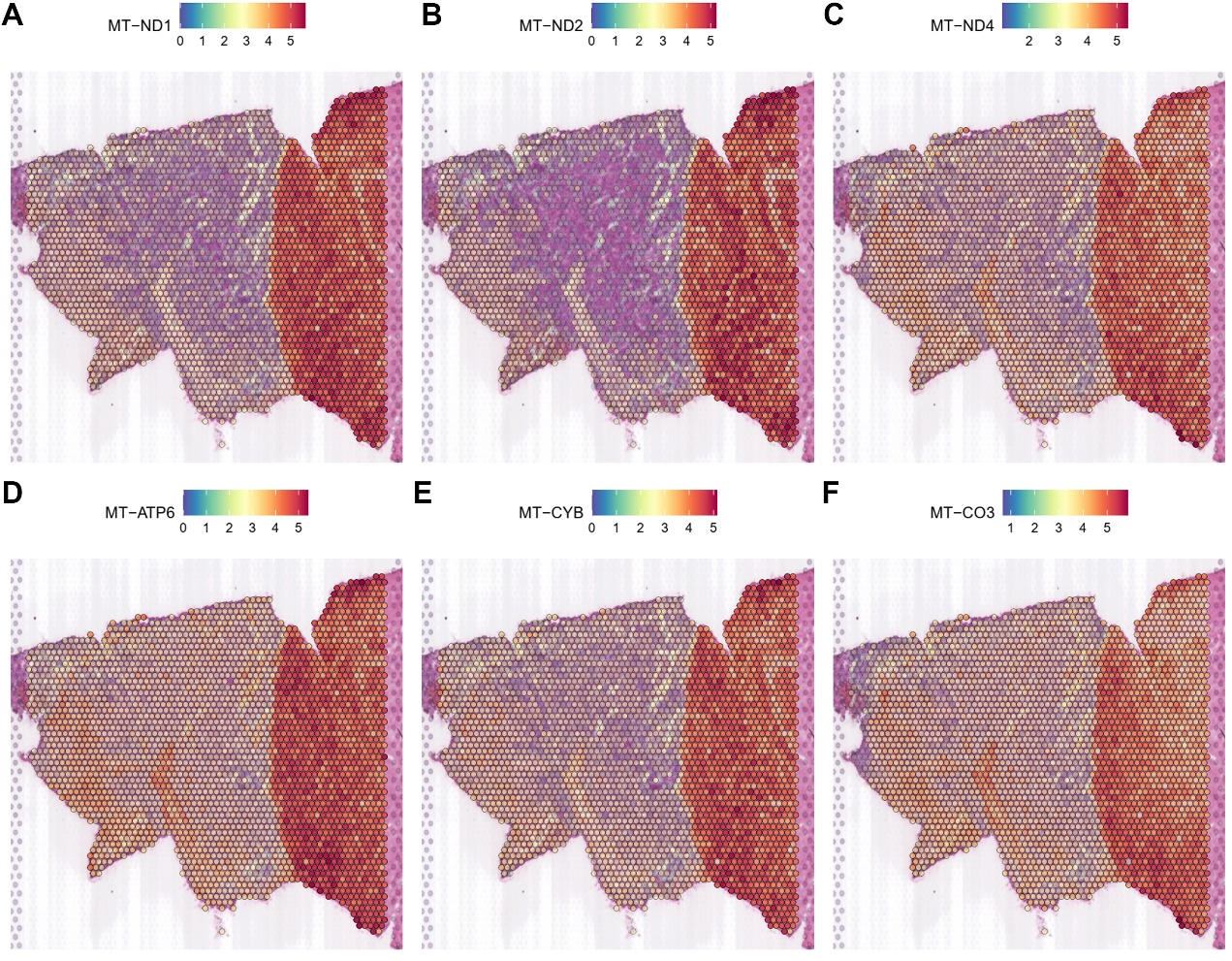


**Figure S21 Expression patterns of mitochondrial genes in HG2 spatial transcriptomics data.** (A-F) The expressions of mitochondrial genes including *MT-CO3, MT-ATP6, MT-CO1, MT-ND1, MT-CYB, MT-ND1* was shown in the ST profile from HG2 tissues.


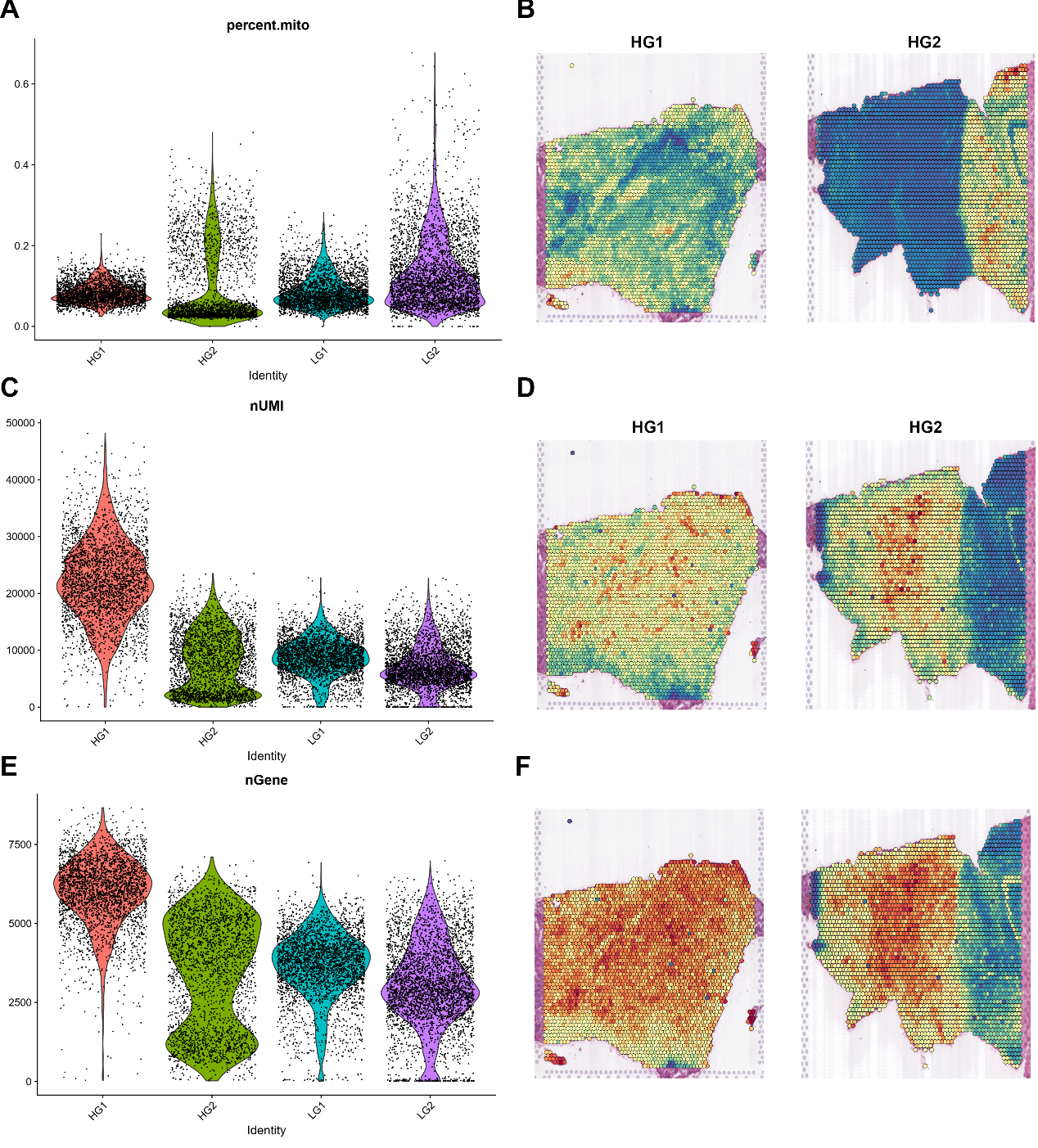


**Figure S22 The loss of mitochondrial genes in specific regions of glioma tissues.** (A) The percentage of mitochondrial gene expressions from total genes was significantly lower in the core regions of HG1 compared to the HG2. (B) The mitochondrial genes were lost in the MES-like and OPC-like malignant regions according to the ST profile from HG2 tissues. (C) The number of UMI in core regions of HG1 compared to the HG2. (D) The UMI in the MES-like and OPC-like malignant regions according to the ST profile from HG2 tissues. (E) The number of genes expressed in core regions of HG1 compared to the HG2. (F) The gene expressed in the MES-like and OPC-like malignant regions according to the ST profile from HG2 tissues.
